# The Representational Consequences of Intentional Forgetting: Impairments to Both the Probability and Fidelity of Long-Term Memory

**DOI:** 10.1037/xge0000128

**Published:** 2016-01

**Authors:** Jonathan M. Fawcett, Michael A. Lawrence, Tracy L. Taylor

**Affiliations:** 1 MRC Cognition and Brain Sciences Unit, Cambridge, United Kingdom; 2 Department of Psychology and Neuroscience, Dalhousie University

**Keywords:** item-method directed forgetting, intentional forgetting, attention, color memory, working memory

## Abstract

We investigated whether intentional forgetting impacts only the likelihood of later retrieval from long-term memory or whether it also impacts the fidelity of those representations that are successfully retrieved. We accomplished this by combining an item-method directed forgetting task with a testing procedure and modeling approach inspired by the delayed-estimation paradigm used in the study of visual short-term memory (STM). Abstract or concrete colored images were each followed by a remember (R) or forget (F) instruction and sometimes by a visual probe requiring a speeded detection response (E1–E3). Memory was tested using an old–new (E1–E2) or remember-know-no (E3) recognition task followed by a continuous color judgment task (E2–E3); a final experiment included only the color judgment task (E4). Replicating the existing literature, more “old” or “remember” responses were made to R than F items and RTs to postinstruction visual probes were longer following F than R instructions. Color judgments were more accurate for successfully recognized or recollected R than F items (E2–E3); a mixture model confirmed a decrease to both the probability of retrieving the F items as well as the fidelity of the representation of those F items that were retrieved (E4). We conclude that intentional forgetting is an effortful process that not only reduces the likelihood of successfully encoding an item for later retrieval, but also produces an impoverished memory trace even when those items are retrieved; these findings draw a parallel between the control of memory representations within working and long-term memory.

Intentional forgetting refers to the effortful updating of memory such that irrelevant or undesirable information is removed or suppressed to reduce the probability of subsequent retrieval. Early paradigms developed to study intentional forgetting in the laboratory often focused on short-term memory (STM), whereas more recent work has emphasized long-term stores (for a review, MacLeod, 1999). Over the last few decades, several paradigms have been developed to address how intentional forgetting arises in different cognitive systems and contexts (e.g., Anderson & Green, 2001; Barnier et al., 2007; Fawcett, Taylor, & Nadel, 2013a, 2013b; Joslyn & Oakes, 2005). These paradigms have been used to understand how intentional forgetting changes during normal aging (e.g., Anderson, Reinholz, Kuhl, & Mayr, 2011; Titz & Verhaeghen, 2010; Murray, Muscatell, & Kensinger, 2011), is impaired through disease or disorder (e.g., Demeter, Keresztes, Harsányi, Csigó, & Racsmány, 2014; El Haj, Postal, Le Gall, & Allain, 2011; Wilhelm, McNally, Baer, & Florin, 1996), and can be employed to suppress memories of trauma (e.g., Catarino et al., 2015; Küpper, Benoit, Dalgleish, & Anderson, 2014) or to maintain a positive self-image by suppressing memories of personal dishonesty (e.g., Shu, Gino, Bazerman, 2011). These paradigms have also been used to reveal interactions of motivated forgetting with attention (e.g., Fawcett & Taylor, 2010; Taylor, 2005; Taylor & Fawcett, 2011) and emotion (e.g., McNally, 2003; Payne & Corrigan, 2007; Quinlan & Taylor, 2014) and the implementation of purposeful forgetting within episodic and autobiographical memory systems (e.g., Fawcett et al., 2013a, 2013b; Joslyn & Oakes, 2005; Noreen & MacLeod, 2014; Stephens, Braid, & Hertel, 2013). Understanding the mechanisms, nature, and limitations of motivated forgetting reveals the ways in which experience and intentions shape our long-term memories and, in so doing, informs clinical (e.g., Blix & Brennen, 2011; Cloitre, 1998; Küpper et al., 2014; Patrick & Christensen, 2013) as well as forensic applications (e.g., Gordon & Connolly, 2010; Kassin & Studebaker, 1998; Pica, Pierro, Belanger, & Kruglanski, 2014; Pica, Pierro, & Giannini, 2015; Thompson & Fuqua, 1998).

## The Mechanisms of Intentional Forgetting

Intentional forgetting can occur during encoding when top-down control is used to limit access to long-term memory stores (e.g., Fawcett & Taylor, 2008; Rizio & Dennis, 2013; Wylie, Foxe, & Taylor, 2008); it can also occur during retrieval when control is engaged to prevent recovery of unwanted traces—including those that escape initial attempts to prevent encoding (for a review, see Anderson & Hanslmayr, 2014). Intentional forgetting that takes place at encoding is of especial interest to us, inasmuch as this stage in the information-processing stream is at the interface of attention, working memory, and long-term memory. The ability to engage top-down control to marshal attentional resources in the service of memory determines which traces are successfully encoded into long-term memory and which are denied access to further processing and storage.

A model technique for studying intentional forgetting at encoding is the item-method directed forgetting paradigm. This paradigm is used to study the mechanisms by which unwanted information is removed from working memory during the encoding epoch and thereby prevented from subsequent retrieval by dint of being weakly encoded into long-term memory (e.g., Thompson, Fawcett, & Taylor, 2011). During the study phase, participants are presented with a series of individual items. Most often these study items are words or pictures (e.g., Quinlan, Taylor & Fawcett, 2010) and each is followed by either an instruction to remember (R) or an instruction to forget (F). Participants must maintain each study item in working memory in anticipation of the memory instruction. Following an R instruction participants engage in elaborative rehearsal of the preceding study item (hereafter: the R item) whereas following an F instruction participants drop the study item (hereafter: the F item) from their rehearsal set. In this way, the R items are selectively rehearsed to the exclusion of F items (e.g., Basden, Basden, & Gargano, 1993). As a result, during a subsequent test phase, participants recall or recognize more R items compared with F items. This difference in memory for R and F items is referred to as a directed or intentional forgetting effect and cannot be better accounted for by demand characteristics (MacLeod, 1999).

Early characterizations of the directed forgetting effect provided no explicit description of the method by which F items are removed from the rehearsal set during encoding. The implication seemed to be that forgetting is attributable to the absence of rehearsal. Yet, despite the explanatory power and intuitive appeal of the idea that forgetting occurs through passive decay of an unrehearsed memory trace, evidence has mounted to suggest that eliminating unwanted F items from the rehearsal set involves one or more effortful processes. For example, Fawcett and Taylor (2008) found that instantiating a study-phase F instruction slowed detection of a subsequent visual probe to a greater degree than instantiating a study-phase R instruction. To the extent that probe reaction times (RTs) provide an index of the relative cognitive demands experienced at the sampled intervals (Kahneman, 1973), the fact that participants were slower to respond following an F instruction than following an R instruction demonstrates that forgetting is not only effortful—in the first seconds of instantiation, it is more effortful than remembering.

Further work has demonstrated an association between intentional forgetting and the withdrawal of visual attention. Taylor (2005) presented study words and localization targets in the right or left visual periphery such that the study words acted as nonpredictive spatial cues for the targets. This arrangement was motivated by the desire to have the study items generate inhibition of return (IOR) for responses to the targets. To the degree that IOR is measurable in target RTs only after attention has been withdrawn from the cued location, Taylor (2005) reasoned that the magnitude of the IOR effect could be used to gauge differential attentional withdrawal following R and F instructions. She observed a larger IOR effect following F compared with R instructions suggesting that participants more readily withdrew attention from a spatial location previously occupied by an F item than from a location occupied by an R item (for related findings and demonstrations that the IOR effect is, in fact, increased following F instructions, see Fawcett & Taylor, 2010; Taylor & Fawcett, 2011; Thompson, Hamm, & Taylor, 2014; Thompson & Taylor, in press).

Taken together, these studies converge on the conclusion that forgetting is an active cognitive process that provides a means of gaining control over the contents of working memory, possibly by shifting internal attention elsewhere. This active view of forgetting does not directly challenge a selective rehearsal account, in that it still attributes much of the directed forgetting effect to the preferential rehearsal of R items, but it is explicit about how the F instruction is implemented to prevent further rehearsal of F items. Rather than characterizing forgetting as the obverse of remembering, this active view of forgetting argues that intentional forgetting is associated with one or more processes that are distinct from remembering (and, therefore, from the failure to remember that defines unintentional forgetting; e.g., Fawcett & Taylor, 2008; Rizio & Dennis, 2013; Wylie et al., 2008).

## The Representational Consequences of Intentional Forgetting

Whatever the processes believed to be responsible, it is now widely understood that intentional forgetting has consequences for both recall and recognition memory. However, in most experiments not all F items are forgotten; some are retained despite task instructions, and differences observed using standard measures of recall or recognition say nothing about the nature of the F item representations that are ultimately retrieved. Nonetheless, the notion that implementing an instruction to forget has consequences for the representation of to-be-forgotten items has precedent. For example, it has been shown that intentional forgetting is observed for measures of recollection, but not familiarity (e.g., Gardiner, Gawlik, & Richardson-Klavehn, 1994). More recently, Fawcett, Taylor, and Nadel (2013a, 2013b) addressed the specificity of to-be-forgotten memories using brief video segments depicting event sequences (e.g., baking cookies). They used specific or general test statements to assess the level of detail with which participants could recollect the content of video segments they had been instructed to remember or forget. Whereas little forgetting was observed for general details of F-instructed events, a robust directed forgetting effect was observed for the specific details of those events. Fawcett et al. concluded that R-instructed information is represented with greater specificity than F-instructed information. Taken together, these findings hint at the possibility that the F instruction impacts the quality of the to-be-forgotten memory trace. However, these studies are not conclusive because their primary dependent measures still fundamentally address the accessibility rather than the fidelity of the relevant information: We know that there are *fewer* recollective experiences for F than R items and that *fewer* details are accessible for F than R episodes. While both of these findings speak to potential differences in the *accessibility* of rich episodic memory traces for F and R items, they do not speak to relative differences in the *fidelity* of those traces that are successfully accessed. As such, the primary goal of the current experiments is to demonstrate that trying to forget reduces the likelihood of F items being retrieved at all, while *also* impoverishing the representations of those items that are retrieved.

## Measuring the Accessibility and Fidelity of Memory

Recent methodological and statistical advances provide an efficient means of distinguishing between trace accessibility and fidelity. For example, Zhang and Luck (2008) report a series of STM experiments in which participants studied a small set of color swatches on each trial (for a precursor to this work, see Wilken & Ma, 2004). Instead of asking participants to make a forced-choice or binary decision (e.g., old/new) to discriminate studied from unstudied items, following the removal of each color swatch and a brief delay, Zhang and Luck (2008) presented a continuous color wheel and prompted participants to indicate the color of one of the swatches they had just studied. The angular distance between the studied and selected colors was then modeled under two different scenarios. Under the first scenario, participants were assumed to have access to the swatch color in memory, such that their responses reflected samples from a von Mises distribution (the circular analog of a normal distribution) that was centered on the studied color within the response wheel. The variability of the response distribution was thus used to denote the fidelity of the corresponding representation, with greater variability denoting poorer fidelity. Under the second scenario, participants were presumed to have no memory of the swatch color such that their responses constituted random samples from a uniform distribution subtending the circumference of the color wheel. Zhang and Luck (2008) observed that whereas the probability of remembering a color decreased rapidly as the number of swatches increased beyond three items, the fidelity with which those colors were represented in working memory was largely unaffected by set size (for further critical discussion, see van den Berg & Ma, 2014).

Particularly relevant to our interest in intentional forgetting, Zhang and Luck (2008) further investigated whether cuing the color swatch that was to be tested on a given trial would increase the fidelity of cued swatches and, conversely, reduce the fidelity of uncued swatches. To achieve this, they modified their study displays to incorporate a line extending from fixation to one of the displayed swatches. On 70% of trials, the cue was valid such that the indicated swatch was tested; on the remaining 30% of trials the cue was invalid, such that an uncued swatch was tested instead. Zhang and Luck (2008) found that encouraging participants to ignore the uncued stimuli reduced both the probability and fidelity of retrieval for uncued items relative to the cued items (see also, Williams, Hong, Kang, Carlisle, & Woodman, 2013).

The approach adopted by Zhang and Luck (2008) is similar in many ways to the short-term variants of intentional forgetting common in the 1960s and 1970s (for a review, see MacLeod, 1999) and provides an interesting connection to modern studies linking intentional forgetting in long-term memory to the control of working memory resources during encoding (e.g., Fawcett & Taylor, 2012). Zhang and Luck’s (2008) findings reveal that ignoring irrelevant stimuli impacts both the probability of forming a STM representation of those items, as well as the fidelity of those representations that are formed. Our question is whether similar impacts on the probability and fidelity of memory occur for long-term episodic memory representations when instructions to remember and forget are used to select items for encoding. If so, the present study will be the first to reveal that memory intentions formed during encoding not only have long-term consequences for the *quantity* of information encoded into long-term memory but also for the *quality* of that information, with an intention to forget leading to *fewer* long-term memory traces of overall *poorer* quality.

## The Current Experiments

The current experiments determined the separate influences that memory instructions have on item accessibility and fidelity in long-term memory. In four experiments we presented colored images representing abstract shapes (Experiments 1–3) or concrete line drawings (Experiment 4), each followed by an instruction to remember or forget. On some trials, a postinstruction visual probe required a speeded detection response (Experiments 1–3). At test, participants were represented with each of the studied items—this time presented in white—as well as an equal number of unstudied foil items.

In Experiment 1, participants made a standard “old”/“new” judgment for each item: This experiment was intended as a “proof-of-concept” to demonstrate that participants could intentionally remember and forget this type of stimulus, which is not easily named (see also Hourihan et al., 2009). In Experiments 2 and 3, two judgments were required at test: An initial “old”/“new” judgment (Experiment 2) or a “remember”/“know”/“no” judgment (Experiment 3), followed by a continuous color judgment. We assessed accessibility by measuring the magnitude of the directed forgetting effect; we assessed fidelity by measuring the deviation between the color in which an image was presented at study and the color selected by the participant from the color wheel presented at test. We restricted our analysis of color accuracy to those trials on which participants self-reported recognition or recollection of the R and F memory traces. To anticipate our findings, we replicated the directed forgetting effect, with more “old” and “remember” responses to R than F items. We also replicated the finding of longer post-F than post-R probe RTs, confirming that intentional forgetting of our complex images is cognitively demanding. Critically, we also obtained evidence of intentional forgetting in our measure of color accuracy, even with performance conditionalized on self-reported memory or recollection of the item in question.

To provide converging evidence for our key findings, in Experiment 4, we removed the initial memory judgment and instead adopted a mixture modeling approach as advocated by Zhang and Luck (2008; Wilken & Ma, 2004) in their study of STM. This allowed us to separate fidelity of a remembered long-term item representation from the probability of that item being remembered, without recourse to subjective judgments of memory. Our modeling results from Experiment 4 corroborated the behavioral results of Experiments 2 and 3 to confirm that instantiating F instructions at encoding not only decreases the probability of subsequently retrieving the to-be-forgotten items from long-term memory, it also reduces the fidelity of those item representations that are retrieved.

## Experiment 1

Prior to addressing our central hypotheses, we first wished to ensure that it was possible to obtain a directed forgetting effect using the abstract visual stimuli we intended to use as study items in Experiments 2 and 3. This was not a foregone conclusion: Most theoretical accounts of intentional forgetting in the item-method paradigm attribute the effect largely to differential rehearsal of those items participants are instructed to remember rather than forget. At least one prior study has observed intentional forgetting using abstract images (Hourihan et al., 2009); even so, we felt that the present stimuli were subjectively less distinct from one another than those that have been used in the past. Therefore, we first presented our stimuli in a standard item-method directed forgetting task using old–new recognition as our dependent measure as a “proof-of-concept” that these stimuli were suitable for our purposes.

Importantly, even if we observed a directed forgetting effect using our complex visual stimuli, we could not be assured that the mechanisms giving rise to greater recognition of R items than F items was related to those that give rise to this pattern with words and nameable objects. To ensure that we could connect our results to the larger literature on item-method directed forgetting, we therefore also included a visual detection probe following 75% of the memory instructions (e.g., Fawcett & Taylor, 2008). As we have noted already, prior research using words has found that instantiating an F instruction enacts a cognitively demanding process associated with a withdrawal of processing resources from the F item representation (Fawcett & Taylor, 2008, 2010; Lee et al., 2013; Taylor, 2005; Taylor & Fawcett, 2011; Thompson & Taylor, in press). This mechanism is presumed to direct attention to other thoughts potentially including (but not limited to) prior items participants had been instructed to remember. Assuming that similar processes are at play when participants are instructed to remember or forget our complex visual images, we predicted slower probe detection RTs following F instructions compared with R instructions.

### Method

#### Participants

Twenty (15 female) Dalhousie undergraduate students participated in this study in exchange for course credit. Participants were naive as to the purpose of the experiment and were tested individually in a session lasting no more than 1 hr. Participants were recruited with an intended sample size of 24, although this value was truncated by the end of the academic term. Intended sample size was determined on the basis of past experiments using the item-method paradigm.

#### Stimuli and apparatus

All experimental procedures were presented using custom software developed in the Python programming language (www.python.org) with the Pygame development library (www.pygame.org) loaded on a 2.8 GHz iMac computer running Mac OS × 10.5. Participants were seated approximately 57 cm from the computer monitor and responses were recorded via a Macintosh USB QWERTY keyboard. All written content, including instructions, was presented against a black background in white, size 18 Gentium Basic Bold (www.sil.org/~gaultney/Gentium/). The memory instructions consisted of two 400-ms tones (high: 1,170 Hz; low: 260 Hz) presented via both channels of Sony MDR-XD-100 stereo headphones.

**Figure 1 fig1:**
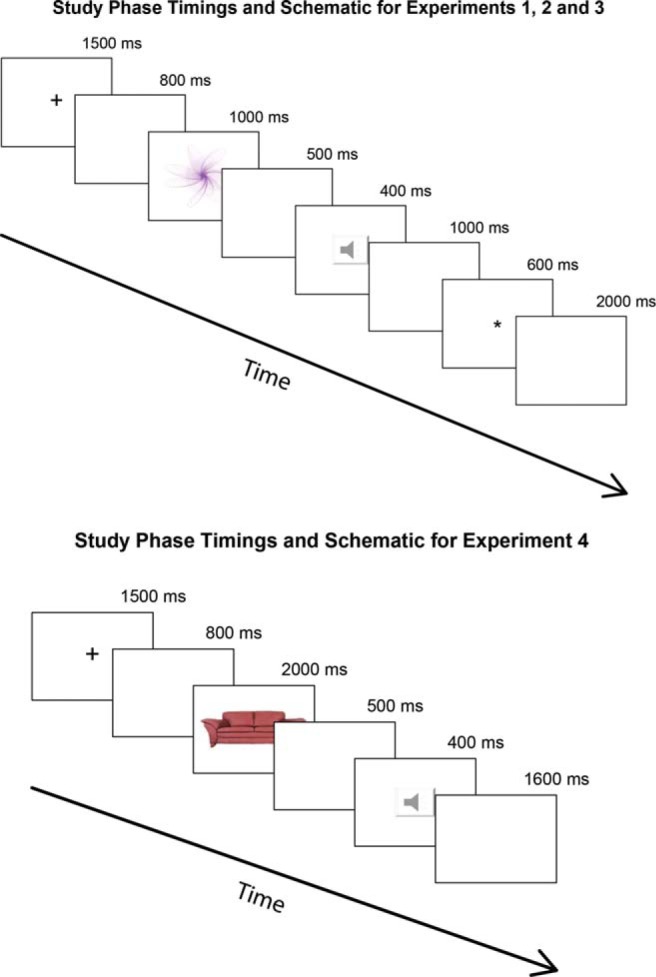
Timings and schematic representation of the study phase trials for Experiments 1–3 (top panel) and Experiment 4 (bottom panel). Experiment 1 used bichromatically presented spiral images; Experiments 2 and 3 used monochromatically presented spiral images (depicted); and Experiment 4 used monochromatically presented line drawings. For Experiments 1–3, probes were present on only 75% of all trials; no-probe catch trials comprised the remaining 25% of all trials. Probes were excluded from Experiment 4. See the online article for the color version of this figure.

Seventy-seven multicolored spiral images (i.e., mathematical roulette curves) were randomly generated by custom software (for an example, see Figure 1). The spirals were rendered to resemble two-dimensional line drawings. Each line was characterized by two random colors to make each image more unique. Prior to running each participant, the custom script used to control the experimental procedures split randomly these renderings into 11 practice items, six buffer items, 15 R items, 15 F items, and 30 foil items. This resulted in a unique combination of items for each participant. Further details on the generation of these stimuli are available from the first author.[Fig-anchor fig1]

#### Procedure

Participants were instructed that they would be presented with a series of abstract images. They were told that they would be asked to remember some of these images whereas others they would be permitted to forget. Half of the participants were instructed to remember all images followed by a high tone and to forget all images followed by a low tone; these designations were reversed for the remaining participants. Participants were also told that a visual detection probe would sometimes follow the memory instruction after a brief delay and that they should respond to this probe as quickly and as accurately as possible. It was mentioned that the memory portion of the experiment (i.e., trying to remember the designated items) was to be their primary focus and that the probe task should be viewed as secondary. Finally, participants were instructed that following the presentation of all study images there would be a test. No mention was made that they would be tested for both R and F items. Once the experimenter was finished providing these verbal instructions the participant was instructed to begin the practice phase during which the experimenter remained in the room.

##### Practice phase

The purpose of the practice phase was to familiarize the participant with the secondary probe task and to introduce the spiral stimuli. Practice phase trials were identical to the study phase trials (see below) except that participants were instructed that they would not be tested for any of the practice items (regardless of memory instruction) and should instead focus on the secondary probe task. Each participant completed a total of 11 practice phase trials under the experimenter’s supervision. Once these trials were complete, the participant was offered an opportunity to ask the experimenter any final questions after which they were left with the written instructions and were told to press enter on the keyboard when ready to begin. The experimenter left the room at this point.

##### Familiarization phase

Prior to beginning the experiment proper, participants were presented with a familiarization phase in which each of the tones and their assigned meaning was presented six times, with the tones intermixed in random order. Concurrent to the presentation of each tone, a brief phrase (“Remember the Image” or “Forget the Image”) describing the intended meaning of the tone was presented onscreen for 3 s.

##### Study phase

As depicted in Figure 1, each trial in the study phase began with the onset of a central fixation stimulus (“+”) lasting 1,500 ms. This stimulus was then removed and the participant was presented with a black screen for 800 ms after which the study item was presented for 1,000 ms. Each study item was drawn randomly without replacement from the 15 R items and the 15 F items. Once the study item was removed, the participant was presented with a black screen for another 500 ms after which the tone that served as the R or F memory instruction was presented for 400 ms during which the screen was black. This was followed by a 600 ms period during which the screen remained black. On probe trials, a visual probe consisting of an asterisk (“*”) was presented for 600 ms; once this probe was removed, participants viewed a black screen for 2,000 ms, after which the trial ended. Participants were instructed to respond to the appearance of a probe as quickly as possible by pressing the spacebar with the index finger of their dominant hand and they were instructed to keep their index finger on the spacebar at all times. No feedback was given. On catch trials the probe was absent but the total trial duration remained unchanged. Therefore, each probe or catch trial lasted for a total of 7,800 ms, which is comparable with past investigations (e.g., Fawcett & Taylor, 2008). The study phase included a total of 30 trials split evenly so that there were 15 R and 15 F trials per participant including 11 probe trials and four catch trials for each level of memory instruction.

Six buffer trials (three at the beginning and three at the end of the study phase) were included to minimize recency and primacy effects. These buffer trials were identical to the study phase trials except that they always included a probe and no RT data were gathered. Buffer images were always followed by an R instruction although recognition performance was not measured for these items.

##### Test phase

Following completion of the study phase, instructions were presented prompting the participant to summon the experimenter. Participants were informed that they would be tested for their memory of the studied items using an old–new recognition task. Participants were told that they would be presented with a series of images, one at a time, and that for each they should indicate whether it had been presented in the preceding study phase. Participants were informed that the test items would include all of the items from the preceding phase regardless of memory instruction and that they should attempt to recognize both R and F items. Responses were made using the “f” and “j” keys: Half of the participants were instructed to press the “f” key to indicate that they recognized the item as “old” and to press the “j” key to indicate that they did *not* recognize the item and believed it to be “new;” these designations were reversed for the remaining participants. Participants were instructed to keep the index finger of each hand on these response keys at all times throughout this phase. Prior to departing, the experimenter asked the participant to repeat these instructions—if unable to do so accurately, the experimenter provided further instruction, otherwise the experimenter left and the test phase began.

During the test phase, participants were presented with the 15 R and 15 F study items randomly interspersed with 30 foil items that had not been presented in any of the preceding phases. Each item was presented individually in the center of the screen until the participant responded. Responses were self-paced and no feedback was provided. Once the test phase was completed, participants were fully debriefed.

#### Statistical tools

We chose to adopt a fully Bayesian approach to analyzing our results. This decision was motivated by several factors, some of which are philosophical and beyond the scope of the present article (for a few examples see Dienes, 2011; Gelman & Hill, 2007; Hoekstra et al., 2014; Kruschke, 2011, 2014). However, two factors are especially germane to analyses of the present data. The first concerns the statistical handling of binary data such as our accuracy measures. Such data are commonly analyzed by aggregating the binary measure into proportions that are then tractable to common statistical techniques (e.g., Analyses of Variance; ANOVAs). Theorists have advised against the analysis of proportions in this manner, instead recommending the use of statistical models that treat the raw binary scores as arising from a binomial distribution (e.g., Dixon, 2008; Jaeger, 2008). Logistic regression is the most common choice, and in the case of designs including a within-subject component (as in our current experiments) multilevel modeling is required. Such multilevel logistic models are readily and efficiently implemented within the *Stan* modeling language (Stan Development Team, 2013), which was our tool of choice. In short, Bayesian modeling provides an adaptive tool with which to optimally represent our data, allowing us to implement multilevel models and to handle non-Gaussian or even complex data structures such as the mixture models described in Experiment 4.

A second motivation pertains to how we interpret the output of our analyses. Bayesian confidence intervals—or in our case, highest density intervals (HDIs; Kruschke, 2014)—provide a direct representation of the most credible values of the estimated parameter after accounting for the (intentionally skeptical) prior beliefs incorporated into the model. As a result, HDIs permit probabilistic statements to be made regarding our confidence that the estimated parameters fall within any particular range. This is similar to the way that researchers often misinterpret Frequentist confidence intervals (e.g., Hoekstra et al., 2014; van de Schoot et al., 2014). Therefore, Bayesian modeling permits us to interpret our results in a manner that is both intuitive and also more rational than common alternatives. It is worth noting here that we have embraced the parameter estimation approach rather than relying upon model comparison (e.g., Bayes Factors) as our primary metric of interest (see Gelman & Hill, 2007; Kruschke, 2010, 2011).

Appendix A provides further details of our statistical approach; recognizing that not all readers will be familiar with this approach, Appendix B provides—where possible—the analogous Frequentist analyses (e.g., ANOVAs) for our core findings. For the reader more interested in our message than the specifics of, or justification for, our analytic technique, our results may be interpreted as any other regression model. We provide the basic parameters relevant to each model (e.g., coefficients) in text, but highlight the comparisons of interest (e.g., comparing R and F conditions) graphically where possible. When interpreting our figures, the median for each condition or contrast is plotted alongside the corresponding HDI for the posterior distribution pertaining to that parameter. As stated above, the HDI represents the values deemed to be most credible for that parameter, given our model and the current data: Therefore, any values falling outside this interval (e.g., 0) are viewed as being unlikely and may be provisionally rejected.

#### Data preprocessing

For Experiments 1, 2, and 3, recognition phase trials for which a response was made in less than 100 ms were rejected on the basis that they were unlikely to reflect actual recognition; the recognition decision times were then log transformed to correct for normality and further trials were rejected if they deviated by more than 3 standard deviations from the overall mean. The intention of filtering our recognition phase data in this manner was to minimize contamination of these judgments by anticipatory responses (i.e., short responses) or distractions (i.e., especially long responses) that could result in the misclassification of trials when conducting our analysis of color judgments in the following experiments. Importantly, inclusion of trials rejected in this manner had no impact on the conclusions reported in any of the following experiments.

### Results and Discussion

In each of our experiments, we present the data from the recognition phase prior to the detection probe data from the study phase as a means of emphasizing their relative importance to our primary hypotheses, rather than their chronology.

#### Recognition phase

Prior to analysis, a total of 0.33% of trials were excluded from all subsequent analyses due to recognition response times that were faster than 100 ms. After applying a log transform to the remaining data, a further 0.25% of trials were excluded from subsequent analyses because they exceeded 3 standard deviations of the overall mean.

Following preprocessing, we fit a logistic multi-level model to retained data by modeling the probability of responding “old” for any given trial as a function of item type (foil, F, R). Coded in this manner, an “old” response corresponds to a “false alarm” for unstudied foil items and a “hit” for the F and R study items. Because item type was a categorical variable, two separate predictors were dummy-coded for the F and R conditions, with foil serving as the relevant intercept. As such, our model estimated three fixed-effect coefficients—the intercept (i.e., the logit transformed proportion of false alarms to foil items) as well as contrasts between this intercept and each of the F and R conditions (i.e., their respective slope coefficients).

**Figure 2 fig2:**
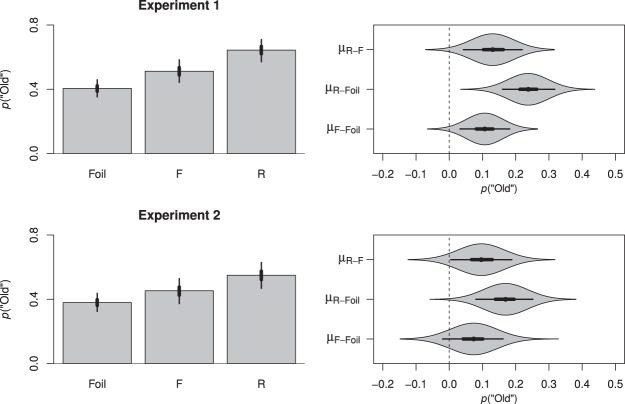
The left column depicts the back-transformed estimated proportion of “Old” responses for Experiments 1 and 2 as a function Item Type (foil, F, R). The right column depicts the pairwise contrasts calculated between each of these conditions; thick lines represent the 50% HDI and thin lines represent the 95% HDI. Polygons depict the posterior distribution for each contrast.

Because our analysis employed logistic regression, the coefficients exist in logit-space. In this metric the intercept was estimated to be −0.39, *HDI*_*95%*_ [−0.61, −0.16], with respective slopes of 0.43, *HDI*_*95%*_ [0.13, 0.74], for F trials and 0.97, *HDI*_*95%*_ [0.64, 1.31], for R trials. To better elucidate our results, the posterior distribution of our model coefficients were combined to produce estimates for each condition and back-transformed into the proportion of “old” responses, as depicted in the top panel of Figure 2. The left frame depicts the predicted back-transformed means for each condition. The right frame further depicts a violin plot of the posterior distributions for the comparisons between each of our conditions (based on the back-transformed values). The point in the center of each polygon represents the median of that difference, the thick lines radiating from this point represent the 50% HDI and the thinner lines represent the 95% HDI. The polygons themselves depict the complete posterior distribution above the point and mirrored below the point. As stated before, to the degree that the 95% HDI for any given contrast (representing the most credible values) does not include 0, it is reasonable to conclude that 0 is not a credible value, given our data.[Fig-anchor fig2]

To summarize the data depicted in Figure 2, we observed a clear directed forgetting effect: Despite the abstract and relatively uniform nature of our study materials, participants were nonetheless capable of intentionally remembering and forgetting according to the relevant memory instructions (see also Hourihan et al., 2009).

#### Study phase

We next analyzed our study phase probe data to determine whether postinstruction probe detection RTs replicated the pattern observed by Fawcett and Taylor (2008). Although longer probe RTs following F than R instructions would not provide conclusive evidence in favor of a common mechanism, it would at least allow for the possibility that intentional forgetting of our complex images is accomplished in a manner similar to the intentional forgetting of words.

Prior to analysis, RTs less than 100 ms were rejected on the basis that they were unlikely to reflect actual detection of the probe; RTs greater than 2,000 ms were automatically excluded because no responses were recorded outside this window. The remaining RTs were log-transformed to correct for non-normality and analyzed as a function of memory instruction (F, R).^1^ This model revealed longer log-transformed RTs for F trials (*M* = 6.34, *HDI*_*95%*_ [6.22, 6.45]) than for R trials (*M* = 6.22, *HDI*_*95%*_ [6.11, 6.33]), with a difference of −0.12, *HDI*_*95%*_ [−0.16, −0.07]. These data are presented in Table 1 alongside the corresponding back-transformed RTs. This finding provides a crucial link between the present paradigm using abstract stimuli and prior work conducted using words: As is true for words, instantiating instructions to forget abstract visual images appears to engage an active, effortful process. [Table-anchor tbl1]

A similar logistic model was also applied to the probability of responding during probe trials, but revealed no difference (intercept = 6.58, *HDI*_*95%*_ [3.98, 10.32]; slope = −1.27, *HDI*_*95%*_ [−4.13, 1.69]), with near-perfect detection accuracy for both F trials (*M* = 99.86%, *HDI*_*95%*_ [98.16%, 100.00%]) and for R trials (*M* = 99.52%, *HDI*_*95%*_ [97.50%, 100.00%]). This finding counters a speed-accuracy trade-off.

We had intended to apply an analogous model to false alarms committed during catch trials if the data permitted this, but only two false alarms were committed across all participants, both during R trials. Because no false alarms were made across any of the F trials, there was no variability and comparisons between these conditions were thus rendered meaningless (the dearth of false alarms is generally true also of Experiments 2 and 3 and therefore we exclude analysis of the probe false alarm data from all of our experiments). We ascribe the relative scarcity of false alarms in the present paradigm to the relatively small number of study items necessitated by the use of our visually complex stimuli and, correspondingly, to the even smaller number of catch trials. Because words are more distinct from one another and therefore do not carry the same risk of floor effects in memory performance as the complex visual stimuli that we employed, similar experiments using words have included as many as 64 R and 64 F items, permitting up to 16 R and 16 F catch trials (e.g., Fawcett & Taylor, 2008). In those experiments, the false alarm rate on probe catch trials was close to 5% for R items and close to 1% for F items. The present design included only four R and four F catch trials, limiting our ability to measure such an infrequent response. Nonetheless, across our experiments the numerical pattern of false alarms supports the trend observed in previous experiments.

## Experiment 2

Having demonstrated intentional forgetting using our abstract line drawings and replicating longer post-F probe RTs than post-R probe RTs, Experiment 2 proceeded to address our primary hypothesis: That intentional forgetting would produce differences in the fidelity with which participants represented those items they had intended to forget but later recognized. To accomplish this we presented the stimuli from Experiment 1 monochromatically: At study, each was presented in a unique color; at test, all were presented in white. Following the initial old–new recognition judgment on each test phase trial, participants were presented with a continuous color wheel and asked to select the color that best approximated the color in which that item had been studied. The absolute angular distance was then computed between the selected color and the studied color to produce an estimate of the fidelity of color memory for that item. To the extent that intentional forgetting influences both the probability of later retrieving an unwanted memory as well as the fidelity of unwanted representations that are later retrieved, we expected color judgments to be less accurate (i.e., larger distance between the selected and the studied colors) for F items than for R items, even after controlling for reported recognition.

Again while not our primary focus, Experiment 2 also included a visual detection probe following 75% of the memory instructions. However, this time we also obtained a within-subjects baseline measure of detection RTs in the absence of a concurrent memory task. By conceptualizing the study-phase probe RTs as deflections from mean performance in this baseline task, we could make more definitive statements regarding whether instantiating an F instruction slows probe detection RTs as we have presumed and/or whether instantiating an R instruction speeds these RTs (e.g., due to increased arousal or attention).

### Method

#### Participants

Twenty-four (18 female) Dalhousie undergraduate students participated in this study in exchange for course credit. Participants were naive as to the purpose of the experiment and were tested individually in a session lasting no more than 1 hr. As in Experiment 1, participants were recruited with an intended sample size of 24 after which recruitment efforts ceased. Although we queried participants about their color vision and excluded no participants on this basis, we did not explicitly test for deficits; doing so was not necessary given that our manipulations were all within-subjects and difficulty with color perception would serve only to increase variability across all conditions.

#### Stimuli and apparatus

The stimuli and apparatus were identical to those used in Experiment 1 with two exceptions. First, only 61 spiral images were generated representing one practice item, 15 R study items, 15 F study items, and 30 foil items. The same image was used for all practice trials and was presented in white. Second, each study item was presented monochromatically in colors sampled equidistantly from a hue-chroma-luminance color wheel. Each individual color was represented only once, although the colors varied continuously from one another and those from proximal regions of the color-wheel were highly similar. Unique color-spiral assignments were used for each participant. Participants were not informed that the colors were manipulated in this manner, or that color would play an important role at test. A separate black-and-white version of each study and foil item was created for use at test.

#### Procedure

The procedure was very similar to Experiment 1, except for the following. First, during the practice phase participants were presented with the same practice image on each trial. Second, following the practice phase (but preceding the tone familiarization phase) we obtained a within-subjects measurement of probe RTs, in the absence of a concurrent memory task. Baseline trials were identical to practice trials in every way (including the use of the same practice image on all trials) except that probe RTs were recorded and catch trials were included. The baseline phase included 12 trials sampled randomly from four probe-R trials, four probe-F trials, two catch-R trials, and two catch-F trials.

Second, in the study phase we eliminated buffer trials. Buffer trials are sometimes—but not always—included as a way of controlling for potential primacy and recency effects. Because we added trials to collect baseline probe RTs, we elected to remove the buffer trials; given that our R and F instructions were randomly determined across trials, there was no reason to believe that primacy and recency would confound our independent manipulation of memory instruction.

Third, several modifications were made to the test phase to incorporate the color selection response. Instead of using the “f” and “j” keys, half of the participants were instructed to respond “a” to indicate that the item was “old” and to respond “s” to indicate that the item was “new,” whereas these designations were reversed for the remaining participants. Participants were instructed to keep the middle and index finger of their left hand on these response keys at all times throughout the test phase. Using the left hand only for the “old”/“new” decision freed the participants’ right hand to make a color response immediately following the old–new response. After making the old–new response, a color-wheel appeared around the perimeter of an imaginary circle that surrounded the test image. A selection cursor appeared at center and participants were instructed to use the mouse to move the cursor and select the color in which the test item had been presented at study; after moving the cursor to the remembered color, the participant clicked the left mouse button to record the selection. The selection cursor disappeared following each selection and always appeared at center following the “old”/“new” response. In the event of a “new” response, participants were instructed to select a color at random. On each trial, the color wheel was rotated by a random value ranging from 0° to 359°. The rotation was intended to avoid any biases in selecting color based on location. All test items were presented in white, which was not represented on the color-wheel.

Finally, following the test phase, participants completed a second probe RT baseline phase. The purpose of the second baseline phase was to obtain a baseline measure of probe RT following extended practice. This phase was presented following the test phase (as opposed to the study phase) to avoid introducing a delay between study and test. There were again a total of 12 such trials, identical to the first baseline phase. Performance on the prestudy and post-test baseline trials were combined for analysis.

### Results and Discussion

#### Recognition phase

Preprocessing revealed no recognition response times faster than 100 ms, so no trials were excluded on this basis. After then applying a log transform, 0.49% of trials were excluded from *all* subsequent analyses because the recognition response times exceeded 3 standard deviations of the overall mean.

##### “Old”-“New” responses

Following preprocessing, accuracy scores were submitted to the same logistic model described for Experiment 1. In this case the intercept was estimated to be −0.49, *HDI*_*95%*_ [−0.73, −0.25], with a slope of 0.30, *HDI*_*95%*_ [−0.08, 0.67], for F trials and 0.69, *HDI*_95%_ [0.32, 1.03], for R trials. To elucidate our findings, the posterior distribution of our model coefficients were again combined to produce estimates for each condition and back-transformed into the proportion of “old” responses as depicted in the bottom panel of Figure 2. In summary, whereas participants were capable of discriminating both R and F items from foil items, this difference was only credible for R items: For F items, 0 was included in the range of credible values, even though 94.26% of the credible values for the comparison between F items and foils fell above 0. The inclusion of 0 in the difference between F and foil items emphasizes the effectiveness of the F instruction for reducing recognition of F items *near* to the level of unstudied foils. In light of this, it is not surprising that our results revealed a significant directed forgetting effect, with better memory for R items compared with F items.

##### Color judgments

Having replicated the directed forgetting effect observed in Experiment 1, we addressed our primary hypothesis: That intentional forgetting would be observed for the color representation of those images that participants recognized, such that participants would show more error in judging the color of the F items that they correctly recognized, compared with the R items that they recognized. After excluding trials based on recognition response times (see above), we thus restricted our analysis of color judgments to those trials on which participants claimed recognition of the study item (i.e., by making an “old” response). This ensured that any differences in the color judgments could not be attributed to overall differences in R and F recognition rates.

**Figure 3:**
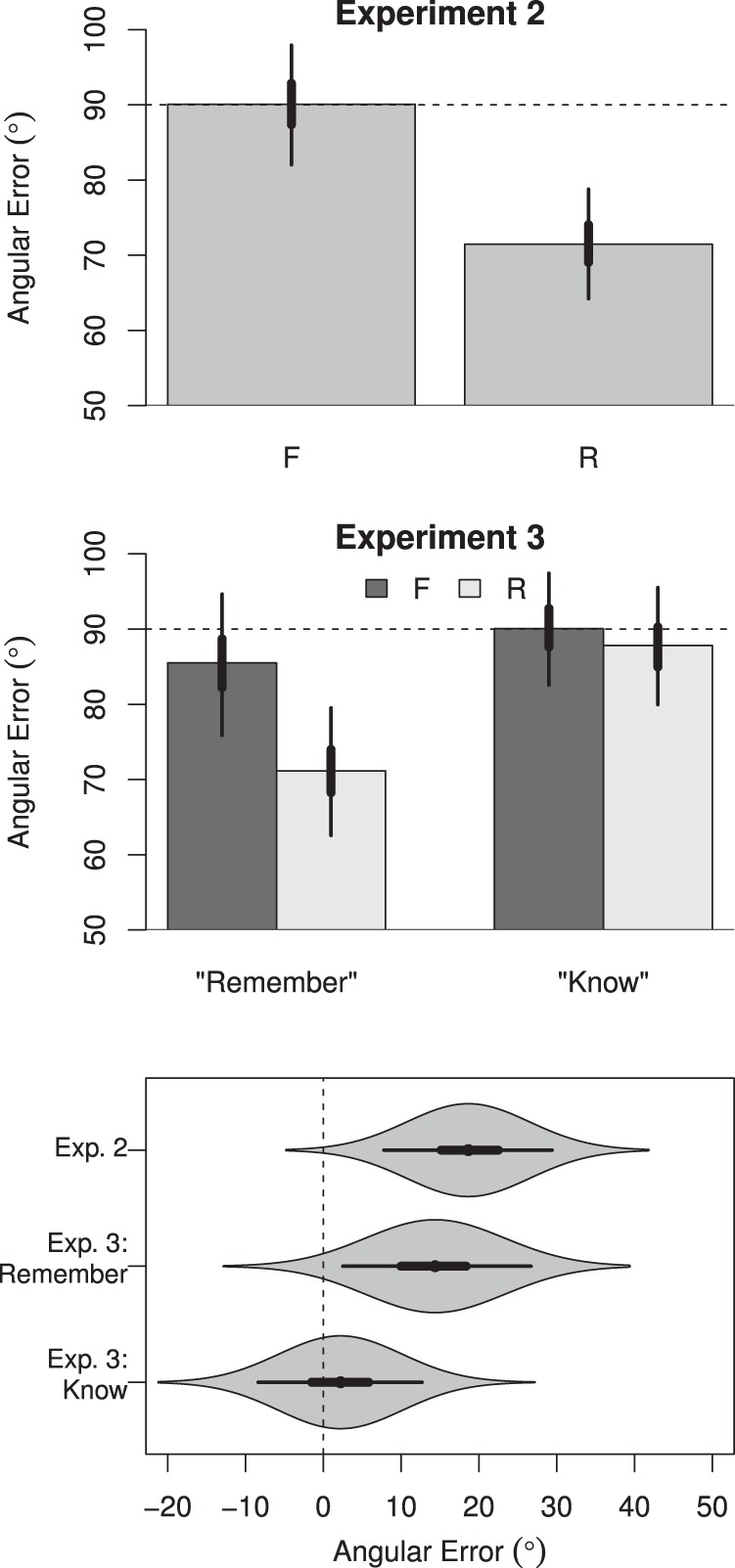
The top and middle panels depict the estimated absolute angular error (in degrees) of color judgments for Experiment 2 as a function of instruction (F, R) and for Experiment 3 as a function of instruction (F, R) and response (Remember, Know). On these plots, chance performance (90°) is denoting by a dashed line. The bottom panel depicts the pairwise contrasts calculated between the R and F conditions for each experiment. In both cases, the thick lines represent the 50% HDI and thin lines represent the 95% HDI. Polygons depict the posterior distribution for each contrast. Note that these data are conditionalized on participants responding “Old” in Experiment 2 or responding either “Know” or “Remember” in Experiment 3.

We operationalized the absolute degrees of error as the angular distance between the color in which an image was studied and the color selected by the participant on the color wheel at test. Using this measurement, 0° reflected perfect performance with a color selection that was identical to the studied item color; 180° represented the greatest error with a color selection that was opposite the studied color on the color wheel; 90° indicated chance performance. This model revealed that participants were less accurate in judging the color of F items (*M* = 90.07°, *HDI*_*95%*_ [82.04°, 97.93°])—for which performance was near-chance—than R items (*M* = 71.45°, *HDI*_*95%*_ [64.22°, 78.78°]), with a difference of −18.70°, *HDI*_*95%*_ [−29.41°, −7.79°]. These data are depicted in Figure 3.^2^

**Figure 4 fig4:**
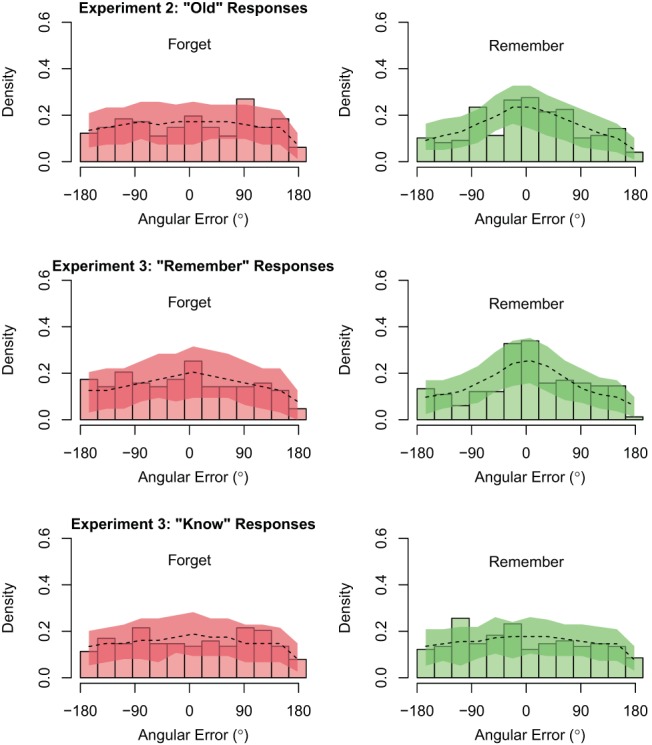
This figure provides a schematic representation of estimated performance derived from the von Mises model fit to the data from Experiments 2 and 3: The histograms depict the distribution of the angular error for the responses within Experiment 2 and 3 whereas the dotted lines depict the median predicted density at each point; the shaded regions represent the 95% HDI of the predicted density at each point. Parameter estimates and pairwise comparisons are discussed in text. See the online article for the color version of this figure.

Whereas the preceding model characterized the effect of instruction as a difference in the mean *absolute* degrees of error, we further modeled the effect of instruction as a difference in the variability of the *relative* or *signed* degrees of error (in this case, ranging from −180° to 180°). This model presumed each response was sampled from a von Mises distribution (the circular analog of a normal distribution) that was centered on the studied color within the response wheel (represented by 0° of error). The variability of the response distribution then denotes the fidelity of the corresponding memory representation, with greater variability denoting poorer fidelity. While perhaps less intuitive, this approach to modeling allows variability in the responses to be interpreted directly in terms of the fidelity of the item representation. Analogous to the preceding models, color judgments were more variable for F items (σ = 135.07°, *HDI*_*95%*_ [113.38°, 158.56°]) than for R items (σ = 99.23°, *HDI*_*95%*_ [85.21°, 116.83°]), with an absolute difference of 35.40°, *HDI*_*95%*_ [10.46°, 61.05°].^3^ These data are depicted in Figure 4.[Fig-anchor fig4]

The fact that participants were less accurate to report the color of F images that they recognized than to report the color of R images that they recognized suggests that memory instructions at encoding impact not only the likelihood of subsequent recognition at test (i.e., the directed forgetting effect) but also the fidelity of those representations that are recognized. In other words, R and F instructions may impact both the *quantity* of information that is encoded to long-term memory and the *quality* of the information that is later retrieved.

To ensure that the differences observed in the color judgments were not driven by a speed-accuracy trade-off, we conducted a comparable analysis of the log-transformed color judgment RTs. Log-transformed RTs were slower for F trials (*M* = 8.40, *HDI*_*95%*_ [8.28, 8.53]) than for R trials (*M* = 8.33, *HDI*_*95%*_ [8.21, 8.46]); while this difference was not quite credible (*M* = −0.07, *HDI*_*95%*_ [−0.16, 0.01]), 95.08% of the credible values for the difference were below 0, supporting a tentative interpretation of slower judgments on F than R trials. Thus, participants’ color judgments of F images were less accurate than for R images *despite* taking somewhat longer to make these judgments.

#### Study phase

As was the case for Experiment 1, we analyzed the study phase probe RTs to establish convergence between the cognitive operations used to instantiate memory instructions for words (e.g., Fawcett & Taylor, 2008) and those used to instantiate memory instructions for our complex images. To do so, we examined the log-transformed study phase probe detection RTs as a function of instruction (F, R) while also accounting for performance in the within-subject baseline phases.

To allow back-transformation into milliseconds—which would not be possible if we modeled performance as a difference from baseline—we conducted a model that included instruction (F, R), task (control, study), and their interaction. The intercept was 6.11, *HDI*_*95%*_ [6.02, 6.19], representing performance for F trials in the control condition; the coefficient for instruction was −0.02, *HDI*_*95%*_ [−0.08, 0.04], representing the difference in performance between F and R trials for the control condition; the coefficient for task was 0.21, *HDI*_*95%*_ [0.15, 0.27], representing the difference in performance between the control and task conditions for F trials. Finally, the interaction term was −0.11, *HDI*_*95%*_ [−0.20, −0.03], representing the degree to which participants were slower for F trials compared to R trials after accounting for baseline speed in the control condition. To summarize the data (also depicted in Table 1), study phase probe RTs were slower than control phase probe RTs regardless of memory instruction—not surprising, given the dual-task nature of the probe task when embedded in the memory task. More importantly, this difference between study phase probe RTs and control phase RTs was especially pronounced for F trials.

As was the case for Experiment 1, the probe RT data are not definitive proof of similarity between the cognitive operations that give rise to a directed forgetting effect for words (e.g., Fawcett & Taylor, 2008) and those that give rise to the directed forgetting effect for our complex images. Critically, however, our probe RT data also do not contradict the possibility of shared operations. This encourages a more general interpretation that memory instructions implemented at encoding influence the quantity and quality of representations in long-term memory, regardless of stimulus type.

Before discussing this conclusion at length, we wish to address two issues that bear on the interpretation of our findings. Past research exploring differences in how R and F words are represented in memory have found R words to be characterized by a greater incidence of recollective experiences and F words to be characterized by greater familiarity in the absence of recollection (e.g., Gardiner et al., 1994). Assuming that this is also true for our complex images, it might reasonably be assumed that color information would only be accessible if the encoding episode were recollected. If so, our observed difference in color judgments for R and F items might arise not directly from differences in the image fidelity as we have presumed, but indirectly from differences in the relative incidence of a recollective experience of the relevant study episode. A related concern stems from the fact that the recognition responses used to parse trials for our analysis of the color judgments are likely to reflect a signal detection process rather than a “pure” measurement of trace accessibility.

Experiment 3 will address these issues by replacing the “old”/“new” recognition response with a “remember”/“know”/“no” response to account for differences in the incidence of recollective experiences. Because “remember” responses are thought by some to reflect high confidence memory judgments (e.g., Yonelinas, 2002), predicating our analysis of the color judgments on those responses should mitigate any concern that the present findings are attributable to differences in the underlying strength of recognized items between our conditions or to differences in the relevant response criterion employed.

## Experiment 3

Experiment 3 was identical to Experiment 2, except that participants made a “remember”/“know”/“no” decision before performing the color judgment at test. If memory instructions influence the fidelity of retrieved image representations, then the color judgments for F images should be more error-prone than the color judgments for R images, even when recognition of both is accompanied by a recollective experience. Such a finding would provide especially strong support for our conclusions.

### Method

#### Participants

Thirty-six (27 female) Dalhousie undergraduate students participated in this study in exchange for course credit. Participants were naive as to the purpose of the experiment and were tested individually in a session lasting no more than 1 hr. We anticipated the need for a larger sample than in our prior experiments to accommodate categorization of test phase color responses as “remember” or “know” rather than simply “old” as was done in Experiment 2. Therefore, participants were recruited with an intended sample size of 36 after which recruitment efforts ceased.

#### Stimuli and apparatus

The stimuli and apparatus for Experiment 3 were identical to those used for Experiment 2.

#### Procedure

The procedure for Experiment 3 was identical to that used for Experiment 2 with one exception. During the test phase, instead of instructing participants to make a yes–no response as in Experiments 1 and 2, participants were instead instructed to make a remember-know-no response. To accommodate the additional response, participants were instructed to rest the ring, middle, and index finger of their left hand on the “a,” “s,” and “d” keys throughout the test phase. They were to press the “a” key to indicate that “no” they did not recognize the item, the “s” key to indicate that they “knew” the item had been presented, and, the “d” key to indicate that they “remembered” the item having been presented. Prior to beginning the test phase conservative instructions were provided (e.g., Rotello, Macmillan, Reeder, & Wong, 2005), wherein participants were given a detailed description of the difference between recollection and familiarity, including examples of each. These procedures produce “remember” and “know” responses that converge with estimates of recollection and familiarity drawn from other sources (see Rotello et al., 2005; Yonelinas, Dobbins, Szymanski, Dhaliwal, & King, 1996; Yonelinas, 2002).

### Results and Discussion

#### Recognition phase

A total of 0.05% of trials were excluded from all subsequent analyses due to response times that were faster than 100 ms. After applying a log transform to the remaining data, a further 3.10% of trials were excluded because they exceeded 3 standard deviations of the overall mean. Separate logistic models were then fit analyzing the probability of remember or know responses as a function of instruction (foil, F, R).

##### “Remember” responses

**Figure 5 fig5:**
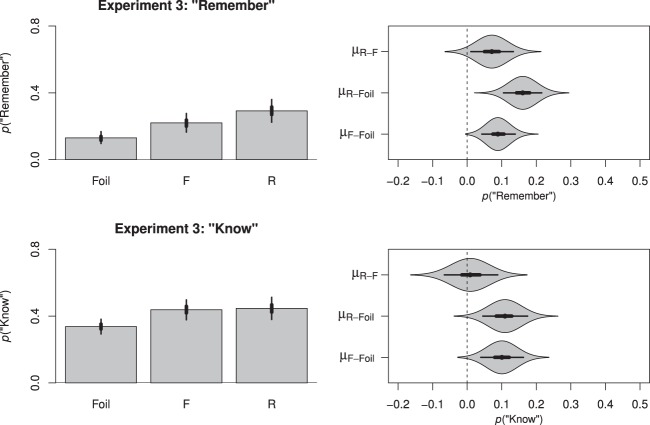
The left column depicts the back-transformed estimated proportion of “Remember” or independent “Know” responses for Experiment 3 as a function Item Type (foil, F, R). The right column depicts the pairwise contrasts calculated between each of these conditions; thick lines represent the 50% HDI and thin lines represent the 95% HDI. Polygons depict the posterior distribution for each contrast. Note that the proportion of “Know” responses is estimated only for those trials not receiving a “Remember” response (e.g., Yonelinas & Jacoby, 1995; see also, Fawcett & Ozubko, 2015).

We first analyzed the probability of participants making a “remember” response, indicating that participants recollected the test item. The intercept for this model was −1.93, *HDI*_*95%*_ [−2.28, −1.62], with a slope of 0.64 for F items, *HDI*_*95%*_ [0.33, 0.94], and 1.02 for R items, *HDI*_*95%*_ [0.69, 1.38]. As depicted in Figure 5, participants were more likely to recollect either R or F items than they were to falsely recollect a foil item and—importantly—in replication of past findings (e.g., Gardiner et al., 1994), participants were also more likely to recollect R items than F items.[Fig-anchor fig5]

##### “Know” responses

Prior to calculating a comparable model for the “know” responses, we had to first address the dependency between “remember” and “know” responses. Because our test phase task required a mutually exclusive response, as the proportion of “remember” responses increased, the proportion of “know” responses necessarily decreased. For this reason, the use of raw “know” responses can underestimate familiarity.

One approach recommended for addressing dependencies in the “remember”/”know” data is to treat these responses independently (e.g., Jacoby, Yonelinas, & Jennings, 1997; Yonelinas & Jacoby, 1995). This can be accomplished by calculating “remember” responses normally but calculating “know” responses as a proportion of remaining trials (i.e., by excluding trials on which a “remember” response was made). We adopted an analog of this approach by limiting our logistic model of “know” responses to those test phase trials on which a “remember” response was *not* made (see also, Fawcett & Ozubko, 2015; for further explanation and proof of equivalence see Appendix A).

When we modeled our resulting “know” data as a function of instruction (foil, F, R), we obtained an intercept of 0.32, *HDI*_*95%*_ [0.28, 0.37], with a slope of 0.61 for F items, *HDI*_*95%*_ [0.55, 0.66], and 0.62 for R items, *HDI*_*95%*_ [0.56, 0.68]. In short, as depicted in Figure 5, whereas participants experienced greater familiarity for F or R items than for foil items, in replication of previous findings (e.g., Gardiner et al., 1994) there was minimal difference in the familiarity of F and R items.^4^

##### Color judgments

After excluding trials based on recognition response times (see above), we further restricted our analysis of color judgments to those trials on which participants claimed to either “remember” or “know” the item. This ensured that any differences in color judgments on R and F trials could not be attributed to differences in the relative incidence of “remember” and “know” responses.

For each trial, we calculated the absolute degrees of error by determining the angular distance on the color wheel between the color in which the test image was presented at study and the color selected by the participant at test; we analyzed error as a function of instruction (F, R) in separate models for “remember” and “know” responses. As a reminder, our strong prediction was that color judgments of “remembered” images would be less accurate for F than for R images. In other words, even when both are associated with a recollective experience (or recognized with high confidence), we expected that the mental representation of F items would show less fidelity than the mental representation of R items. We made no explicit prediction for images associated with a “know” response, given that a feeling of familiarity neither necessitates nor discounts access to a detailed mental representation.

Our results showed that color judgments following “remember” responses were less accurate for F items (*M* = 85.48°, *HDI*_*95%*_ [76.91°, 95.38°]) than for R items (*M* = 71.05°, *HDI*_*95%*_ [63.20°, 79.69°]), with a difference of −14.40°, *HDI*_*95%*_ [−27.24°, −2.98°]. However, for color judgments that followed “know” responses, accuracy was roughly equivalent for F items (*M* = 90.20°, *HDI*_*95%*_ [82.25°, 97.44°]) and R items (*M* = 87.94°, *HDI*_*95%*_ [80.14°, 95.16°]), with a difference of only −2.23°, *HDI*_*95%*_ [−13.13°, 8.11°]. These data are depicted in Figure 3.

The same pattern emerged when the data were instead modeled as arising from a von Mises distribution. Color judgments following “remember” responses were more variable for F items (σ = 131.33°, *HDI*_*95%*_ [108.67°, 158.53°]) than for R items (σ = 102.90°, *HDI*_*95%*_ [84.23°, 125.92°]), with a difference of 27.64°, *HDI*_*95%*_ [2.73°, 53.84°]. However, following “know” responses, variability in color judgment responses was roughly equivalent for F items (σ = 171.96°, *HDI*_*95%*_ [129.27°, 225.07°]) and R items (σ = 171.42°, *HDI*_*95%*_ [125.12°, 223.33°]), with a noncredible difference of 1.08°, *HDI*_*95%*_ [−32.58°, 34.94°]. These data are depicted in Figure 4. In short, memory instructions revealed an effect on the fidelity of an image representation even on trials for which participants reported access to the original encoding episode. This undermines an alternative explanation that the effects of memory instruction on fidelity in Experiment 2 were driven by differences in the relative incidence of R and F item recollection.

To determine whether the accuracy of the color judgments traded for speed, we also analyzed the log-transformed color judgment RTs. For color judgments that followed “remember” responses, participants exhibited comparable log-transformed RTs for F items (*M* = 8.49, *HDI*_*95%*_ [8.36, 8.61]) and R items (*M* = 8.47, *HDI*_*95%*_ [8.35, 8.59]), with a minimal difference of −0.02, *HDI*_*95%*_ [−0.10, 0.08]. The fact that color judgments were made with approximately the same speed across our conditions implies that these responses were roughly equated with respect to the confidence with which they were made. Interestingly, even when thus equated, a difference in fidelity was still observed. This finding supports prior evidence suggesting that differences in recollective detail are possible even after accounting for differences in memory strength (e.g., Ingram et al., 2012).

Unlike color judgments that followed “remember” responses, color judgments that followed “know” responses were slightly slower for F items (*M* = 8.56, *HDI*_*95%*_ [8.45, 8.68]) than for R items (*M* = 8.46, *HDI*_*95%*_ [8.35, 8.57]), with a difference of −0.10, *HDI*_*95%*_ [−0.18, −0.01]. The trade-off of speed and accuracy for color judgments that followed “know” responses suggests that participants might have been more confident judging the color of R images than the color of F images. We have no explanation for why this might be true. In any case, our conclusion remains the same: The effect of memory instruction on the accuracy of a subsequent color judgment is not attributable to differences in the incidence of reported recollection.

#### Study phase

As in the previous experiments, we examined the study phase probe RT data to determine whether the pattern of longer post-F RTs than post-R RTs can be replicated using complex images as stimuli for the memory task. As we did for Experiment 2, we log-transformed study phase RTs and modeled them as a function of instruction (F, R), using performance in the within-subject baseline tasks as a control. In this case, the intercept was 6.01, *HDI*_*95%*_ [5.95, 6.06], representing performance for F trials in the control condition; the coefficient for instruction was −0.02, *HDI*_*95%*_ [−0.07, 0.03], representing the difference in performance between F and R trials for the control condition; the coefficient for task was 0.22, *HDI*_*95%*_ [0.16, 0.29], representing the difference in performance between the control and task conditions for F trials. Finally, the interaction term was −0.09, *HDI*_*95%*_ [−0.17, −0.01], representing the degree to which participants were slower for F trials compared with R trials after accounting for baseline speed in the absence of a concurrent memory task. These data confirm that study phase probe RTs were again slower than control phase probe RTs—and that this difference was larger for F trials than for R trials. These data are depicted in Table 1.^5^

## Experiment 4

Experiments 2 and 3 provide compelling evidence that memory instructions impact the fidelity of a retrieved memory representation, even after accounting for self-reported recognition (Experiment 2) and recollection (Experiment 3). However, we have thus far relied upon our participants to distinguish between those items they recognized or recollected and those they did not. While we believe our participants capable of these distinctions, it is nevertheless possible that on some trials they responded incorrectly—ostensibly recognizing or recollecting an item for which they could not, in fact, retrieve a representation or encoding episode. Reliance on self-reported recognition or recollection also ignores possible differences in the relative strength of the memory trace or response criterion employed in making those memory judgments. Furthermore, reporting a recollective experience of an encoding episode implies, but does not necessitate, access to the color of the image presented in that encoding episode. To address these concerns, our final experiment used a mixture-modeling technique to parse the effect of memory instructions on the *probability* of retrieving the color for a given item from their effect on the *fidelity* of the resultant representation.

As discussed earlier, our chosen technique has become popular in the visual short-term working memory literature (e.g., Lawrence, 2010; Zhang & Luck, 2008) and involves modeling continuous color judgments for a recently studied color swatch as arising from two separate scenarios. For some portion of responses, participants are presumed to have access to the swatch color in memory and to thus produce a response sampled from a von Mises distribution (the circular analog of a normal distribution) that is centered on the studied color within the response wheel. Under this scenario, the variability of the response distribution denotes the fidelity of the corresponding memory representation (i.e., with greater variability denoting poorer fidelity). For the remaining responses, participants are presumed to have no memory of the swatch color and must therefore guess, producing a response that is sampled from a uniform distribution subtending the circumference of the color wheel.

Conceptualizing the underlying response distributions in this manner, there are two parameters of interest that broadly correspond to the two test phase measures included in Experiments 2 and 3. First, this modeling approach estimates the probability that participants have some memory of the studied color (ρ or the *probability* of memory): This value most closely corresponds to our measures of recognition and recollection. The second parameter of interest corresponds to the precision of the von Mises distribution from which responses are sampled (κ or the *fidelity* of memory): This parameter represents the variability with which the color is represented in memory when present; this parameter is thematically similar to our conditionalized analyses of the color judgments from Experiments 2 and 3. Note that in discussing these terms with respect to our Experiment 4 data, we will adopt the convention of converting κ (precision) to σ (standard deviation) using the formula provided by van den Berg and Ma (2014);^6^ we believe this to be advantageous because σ is more commonly encountered in cognitive science, making its interpretation more intuitive.

On the basis of Experiments 2 and 3, we predicted an effect of memory instruction on both ρ and σ. This prediction is further supported by supplementary evidence that forcing a putatively irrelevant color swatch out of working memory decreases ρ and increases σ in a short-term memory task (Williams, Hong, Kang, Carlisle & Woodman, 2013; Zhang & Luck, 2008) and should thus have similar down-stream implications for the formation of long-term memories. However, it is worth noting that the viability of this modeling approach for color judgments has only recently been demonstrated in the context of a more typical long-term memory experiment with many items and at long study-test delays (Brady, Konkle, Gill, Oliva, & Alvarez, 2013). As such, despite a strong basis for our predictions we were nonetheless motivated to modify our paradigm to further maximize overall memory performance and therefore our odds of resolving instruction-related differences in our parameters.

We eliminated the study phase detection probe from our design. After repeated demonstrations that we could replicate the pattern of longer post-F than post-R probe RTs that has been reported for word stimuli (Fawcett & Taylor, 2008), further replication was not deemed central to our primary objective. To ensure an adequate number of trials to conduct our model, we substantially increased the number of stimuli—from 15 to 60 items per condition. Because we felt that such a large number of abstract stimuli would surely overwhelm our participants’ attempts to intentionally commit half the study items to memory, we instead presented line drawings generously provided by Brady, Konkle, Gill, Oliva, and Alvarez (2013). Finally, we dropped the initial recognition judgment from the test phase—requiring participants to make only a color judgment for each test item. This change made the inclusion of foil items unnecessary and also ensured that the color judgment was not influenced by a prior memory report.

### Method

#### Participants

Thirty-three (25 female) Dalhousie undergraduate students participated in this study in exchange for course credit. Participants were naive as to the purpose of the experiment and were tested individually in a session lasting no more than 1 hr. Although the basic design of the present experiment was simpler than those preceding it, we anticipated the need of a relatively large sample to permit our intended modeling approach. Therefore, participants were again recruited with an intended sample size of 36, although this value was truncated by the end of the academic term.

#### Stimuli and apparatus

The stimuli and apparatus were identical to the preceding experiments with the exception of our stimuli: For our study materials, 120 line drawings were sampled randomly from those used by Brady et al. (2013; downloaded from http://timbrady.org/resources.html). These drawings were assigned a unique color at study in the manner described for Experiments 2 and 3, resulting in a total of 120 colored images.

#### Procedure

The procedure for Experiment 4 was identical to the procedure used in Experiment 3 with the following exceptions.

We made four changes to the study trials. First, we increased the total number of study phase trials from 15 replications per memory instruction (30 in total) to 60 replications per memory instruction (120 in total). Second, to accommodate the larger number of study trials, we increased the study item presentation time. Our rationale for doing so was based on the fact that participants were instructed to intentionally commit half of the study items to memory; even though intentional forgetting is generally improved as cognitive load increases (Lee & Lee, 2011), we did not want the larger number of study trials to seem overwhelming and to cause our participants to disregard the memory instructions. As such, study phase trials now began with a 1,500 ms fixation stimulus followed by an 800 ms pause and then the study item for 2,000 ms. Third, to accommodate the longer study item presentation time combined with the larger number of study trials, we removed the probe RT task and the complementary baseline RT task. Thus, upon removal of the study item, participants again viewed a blank screen for 500 ms at which point the memory instruction was presented for 400 ms and then a 1,600 ms inter-trial interval was observed. This resulted in a total study trial duration of 6,800 ms; these timings are depicted in Figure 1.

We made two changes to the test trials. First, we excluded a memory report, such that no “old”/“new” or “remember”/“know”/“no” response was elicited to test stimuli. Instead, each test phase trial presented a study item and participants were required only to select the color in which that item had been studied. This change also meant removing all the foil items from the test phase, resulting in 60 R and 60 F test phase trials (120 trials in total). Second, we modified the color selection task to approximate the procedure used by Brady et al. (2013): As before, participants used the mouse to move a selection cursor along a color wheel whose perimeter circled each test item—however, instead of presenting the study item in white throughout the trial, the color of the test image updated to reflect the color indicated by the selector. We surmised that this change might allow participants to better “tune” their responses prior to selection by directly comparing the colored test stimulus to their stored mental representation.

### Results and Discussion

The only measure collected in this experiment was the color judgment made for each test item. These responses were submitted to a multi-level mixture model estimating ρ (the probability of memory) and κ (the fidelity of memory); however, as mentioned earlier κ was then converted to σ, which is what we report. Further details are available in Appendix A, with variants also described by Lawrence (2010) and Zhang and Luck (2008).

Recognizing recent critiques of extant modeling approaches, we incorporated variability in encoding precision into our estimates of σ (e.g., van den Berg, Awh & Ma, 2014; van den Berg, Shin, Chou, George & Ma, 2012). Inclusion of this term was supported by modest improvements in out-of-sample prediction accuracy as measured by the Watanabe-Akaike Information Criterion (*WAIC*_*FP – VP*_ = 6.6, *SE* = 2.4) and by Leave-One-Out cross-validation with Pareto Smoothed Importance Sampling (*LOO-PSIS*_*FP – VP*_ = 4.8, *SE* = 2.6; Vehtari, Gelman, & Gabry, 2015). For this reason, we have chosen to present only the parameters from the best-fitting variable-precision mixture model. Note that adopting a more common fixed-precision variant (e.g., see Table 2 of van den Berg et al., 2012) did not change any of the reported conclusions.^7^

These metrics (ρ and σ) were each modeled as a function of instruction (F, R), with the prediction that memory instruction would influence both. This prediction was supported. For ρ, our model indicated a higher back-transformed probability of recollecting R items (*M* = .65, *HDI*_*95%*_ [.59, .72]) compared with F items (*M* = .51, *HDI*_*95%*_ [.43, .59]), with a difference of .14, *HDI*_*95%*_ [.07, .22]. This finding corresponds to the directed forgetting effect as measured by more typical recognition or recall tasks such as those used in Experiments 1, 2, and 3: Items participants were instructed to remember were more likely to be accessible at test than items participants were instructed to forget. When viewed in comparison with the current Experiment 3, the effect of instruction on the probability of memory also links ρ to estimates of reported recollection; this follows from the observation that intentional forgetting implemented at encoding generally only occurs for recollection (e.g., Gardiner et al., 1994).

**Figure 6:**
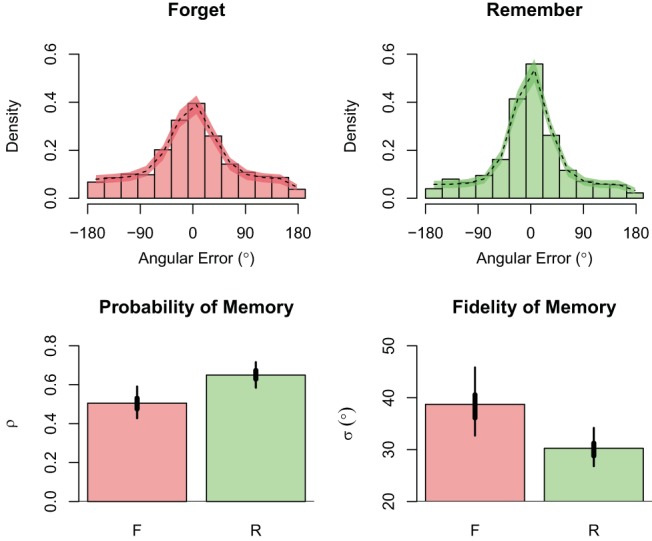
The top panel of this figure provides a schematic representation of estimated performance in the mixture model fit to the data from Experiment 4: the histograms depict the distribution of the angular error for the responses within Experiment 4 whereas the dotted lines depict the median predicted density at each point; the shaded regions represent the 95% HDI of the predicted density at each point. The bottom panel of this figure depicts the back-transformed probability that participants retrieved the studied color (ρ) and the fidelity (in degrees) of the representation of those colors that were retrieved (σ) as estimated from the mixture model conducted on the data from Experiment 4; thick lines represent the 50% HDI and thin lines represent the 95% HDI. Pairwise comparisons are discussed in text. See the online article for the color version of this figure.

A complementary pattern was observed for σ, with greater variability observed for F items (*M* = 38.73°, *HDI*_*95%*_ [32.72°, 45.82°]) than for R items (*M* = 30.24°, *HDI*_*95%*_ [26.81°, 34.16°]), resulting in a difference of 8.45°, *HDI*_*95%*_ [2.51°, 15.07°]. These findings provide final converging evidence of our hypothesis that memory instructions impact the fidelity of the image representation that is formed for R and F items. These data are also depicted in Figure 6.^8^

## General Discussion

Across four experiments using two rather different approaches, we provide the first clear evidence that instructions to remember or forget at encoding impact both the probability of subsequently retrieving those item representations from long-term memory and the fidelity of those representations that are retrieved. To wit, images that were followed by an F instruction during encoding were less likely to be successfully retrieved from long-term memory than images that were followed by an R instruction. And even when images were successfully recognized at test, memory for the color of the studied images was worse if those images had been F-instructed at study rather than R-instructed. This effect of memory instruction on the fidelity of memory was pronounced when retrieval was accompanied by a recollective experience, arguing against the effects on fidelity being a by-product of fewer recollections of F than R episodes. Importantly, our key findings were replicated using a modeling approach that did not depend on subjective self-report to separate the items that were remembered from those that were not.

We begin by discussing the implications of our findings with regard to how we understand the mechanisms and consequences of intentional forgetting. We end by discussing how these findings relate to other paradigms, recent advances in our understanding of the control of working memory, and also how these findings might impact our understanding of intentional forgetting in applied contexts.

### The Mechanisms and Representational Consequences of Intentional Forgetting

The critical message from the current experiments is that the effects of memory intentions formed at encoding are not limited to variation in the probability of retrieving study episodes from long-term memory: Memory intentions formed at encoding also influence the fidelity of those episodes that are later retrieved. While memory performance was not stellar in any condition within our first three experiments, this is attributable to the difficulty of our memory tasks. Task difficulty resulted from the complexity and visual similarity of the abstract stimuli that participants were instructed to intentionally commit to memory during the study phase; task difficulty was compounded by the surprise color judgment task for which participants could not prepare.

Despite the difficulty of the tasks, we replicated the typical directed forgetting effect by showing greater overall recognition and recollection of R compared with F items. We also demonstrated that the pattern of longer probe RTs following F instructions than following R instructions (see Fawcett & Taylor, 2008) replicates when complex visual images are employed as study items rather than words. And, most importantly, we showed that even when general recollection of F items persists at levels strong enough to drive successful recognition (contravening the intention formed at encoding), the details of those memory representations are still impoverished relative to R items. Inspection of Figures 3 and 4 reveal that color judgments for recognized or recollected F items were only slightly better than chance performance (i.e., 90°) and Figure 6 clearly demonstrates that the item representations that participants did successfully retrieve were more variable at test for F items relative to R items. This was true whether participants self-reported their memory of R and F items (Experiments 2 and 3) or whether the probability of accessing the color in memory was estimated mathematically and independently of the participants’ subjective report of recognition/recollection (Experiment 4).

The finding that probe detection responses were slower following F instructions than R instructions suggests that attempting to intentionally forget complex images is initially more cognitively demanding than attempting to commit them to memory. Past research has postulated an active cognitive mechanism associated with forgetting unwanted words (e.g., Fawcett & Taylor, 2008) that has also been found to interact with attentional orienting (e.g., Fawcett & Taylor, 2010; Taylor, 2005; Thompson et al., 2014; Thompson & Taylor, in press), reduce color-naming interference (Lee et al., 2013) and interfere with incidental memory formation (Fawcett & Taylor, 2012). Together, these findings implicate the effortful withdrawal of attention from the representation of the to-be-forgotten information (as well as other spatially and temporally proximate information) in the brief period following an F instruction. The consequence of this hypothesized process is to terminate covert rehearsal (e.g., Hourihan & Taylor, 2006), with the presumed goal of removing the F item from working memory and ultimately liberating limited-capacity attentional resources for other purposes, including rehearsal of prior R items.

We believe that the effect of memory instruction on study phase probe detection responses in the current experiments reflects the engagement of a similar active mechanism associated with limiting the commitment of unwanted images (F items) to memory. This belief is bolstered by recent evidence that forcing a putatively irrelevant color swatch out of working memory similarly diminishes ρ and increases σ in a short-term memory task (Williams et al., 2013; Zhang & Luck, 2008). The expulsion of F items from working memory and the consequent or coincident selective rehearsal of R items could account for the weaker, more impoverished representation of F items within long-term episodic memory and the correspondingly stronger, more accurate representation of R items. This makes apparent that control processes engaged to cease rehearsal of active traces within working memory shape the contents of long-term memory by influencing not only which items are ultimately represented but also the quality of those representations. It seems likely that these early influences of encoding intentions are supplemented as necessary by other processes that likewise operate to restrict long-term memory formation from within working memory or that operate directly on the long-term memory representations to further shape their content and character. Indeed, we posit that control over encoding represents just one of many possible ways in which we can influence the contents of our own memories.

Interestingly, recent neuroimaging studies have demonstrated that intentional forgetting performed at encoding recruits regions of the right prefrontal cortex (rPFC) that are implicated in the down-regulation of hippocampal activity that is otherwise critical for the formation of new memories (Ludowig et al., 2010; Rizio & Dennis, 2013). We know that ambient hippocampal activity in the period surrounding an event epoch predicts subsequent memory for that event (e.g., Davachi et al., 2003; Park & Rugg, 2010). By extension, the down-regulation of the hippocampus following an F instruction would be expected to limit encoding of the episode in which the F item was embedded. This might provide an additional avenue through which unwanted memories are ultimately forgotten—and might likewise explain the disruption of incidental memory formation observed following instructions to forget (Fawcett & Taylor, 2012; see also, Hulbert & Anderson, 2011). However, we would argue that the down-regulation of hippocampal activity alone is unlikely to explain the interactions observed between intentional forgetting and attentional orienting (e.g., Fawcett & Taylor, 2010; Taylor, 2005; Thompson et al., 2014; Thompson & Taylor, in press), which might be related instead to modulation of regions in the parietal cortex (for a review of the neuroimaging data, see Anderson & Hanslmayr, 2014). Our current belief is that a full account of intentional forgetting requires the withdrawal of attention away from the representation and rehearsal of unwanted memories, possibly followed by an active suppression of the encoding epoch to limit both the probability and fidelity of subsequent retrieval.

This is not to claim that the attentional and mnemonic mechanisms purported to underlie intentional forgetting operate in isolation. Recent research exploring competition between item representations within working memory provides one possible avenue through which the incomplete withdrawal of attention from an unwanted item in working memory could trigger suppression of the resulting memory trace. According to the nonmonotonic plasticity hypothesis (e.g., Detre, Natarajan, Gershman, & Norman, 2013), suppression occurs when a moderately activated representation competes for attentional focus against another, more strongly activated representation in working memory. For example, trying to remember the content of one’s grocery list while concurrently retrieving directions to the nearest supermarket would place these concepts into competition. To the extent that the directions “win” attentional focus, memory for the directions would be strengthened while memory for the items to be purchased would be weakened. However, as the name implies, the relationship between activation and suppression is nonmonotonic—and concepts that are only weakly activated (e.g., the amount of money available to purchase the required groceries) are unaffected. As such, this hypothesis argues that changing from one thought to the next can at times weaken the representation of the preceding thought, depending on whether that thought remains moderately active (relative to the activation level of the new thought) in working memory.

To test this hypothesis, Lewis-Peacock and Norman (2014) provided participants with two pictures on each trial, with the instruction to focus on one and ignore the other. Importantly, following the disappearance of both stimuli, participants were sometimes instructed to switch their internal attentional focus to the previously unattended picture. Because the pictures were no longer on-screen, this operation was necessarily enacted entirely within working memory. Using pattern classifiers applied to functional magnetic resonance imaging data acquired throughout the task, these researchers then tracked the activation of the attended and unattended pictures during the switch trials with the intention of relating the degree of competition (i.e., co-activation) between the two pictures following the switch to performance on a surprise memory test for the formerly attended items. Supporting the predictions of the nonmonotonic plasticity hypothesis, competition between the two items was predictive of worse memory for the formerly attended items; however, performance for the formerly attended items was relatively unimpaired if those items were either weakly or especially strongly activated following the switch.

In light of these findings, the withdrawal of attention from an unwanted item could place that item representation into a losing competition with some other thought—such as the rehearsal of the preceding R items in the case of an intentional forgetting paradigm (c.f. Lewis-Peacock & Norman, 2014). If so, some portion of the difference in memory performance for R and F items could be attributable to suppression arising from the dynamics of competition resolution in working memory. In this regard, we view the present experiments—as well as much of our recent research—as fundamentally concerned with the cognitive mechanisms and consequences associated with exerting control over the contents of working memory (e.g., Fawcett & Taylor, 2012). Therefore, we would argue that the same active mechanisms hypothesized in the present experiments are likewise at play in similar experiments dealing with short-term memory (e.g., Williams et al., 2013; Zhang & Luck, 2008), possibly explaining the similarity in our behavioral and modeling outcomes. Nonetheless, the extent to which these processes are identical in their implementation and consequences is a question for future research.

The discussion of mechanisms also raises the question as to whether other forms of intentional forgetting with different underlying processes would likewise affect both the probability and fidelity of memory. Although we hesitate to generalize too broadly to all other mechanisms of intentional forgetting, we do believe that our current findings will be generalizable to the think/no-think paradigm. In the think/no-think paradigm (for reviews, see Anderson & Hanslmayr, 2014; Anderson & Huddleston, 2012), participants are trained on a list of cue-target pairs until the cue reliably reminds the participants of the associated target. The cues are then presented during an additional phase without their corresponding target and participants are instructed to either bring the target to mind (think trials) or to suppress retrieval of the target (no-think trials). The think/no-think paradigm shares a great deal of thematic and theoretical overlap with the current item-method task: Both require control over memory, in the current case at encoding and in the think/no-think paradigm at retrieval; both tasks are associated with impaired incidental memory formation following a forget/no-think instruction (Fawcett & Taylor, 2012; Hulbert & Anderson, 2011); and, both tasks draw upon a common neural network, including regions of the dorsolateral prefrontal cortex that have been found to down-regulate activity in the hippocampus following a forget/no-think instruction (Anderson et al., 2004; Hulbert & Anderson, 2011; Wylie et al., 2008). Interestingly, both tasks likewise appear to involve parietal regions thought to play a role in attentional orienting (Anderson & Hanslmayr, 2014). However, whereas the think/no-think paradigm is postulated to invoke both a proactive control mechanism to suppress the entry of unwanted information into conscious awareness, followed if needed by a reactive control mechanism to expunge any unwanted information from attentional focus (Anderson & Huddleston, 2012; Fawcett et al., 2015), we would argue that the item-method directed forgetting task involves only the latter process (Fawcett & Taylor, 2012). It is this reactive process of expunging an unwanted F item from working memory that we have argued is cognitively demanding (e.g., the present study phase probe RTs; see also, Fawcett & Taylor, 2008), interacts with the orienting of visual attention (Fawcett & Taylor, 2010; Taylor, 2005) and ultimately terminates rehearsal (Hourihan & Taylor, 2006) to facilitate the removal of unwanted information (Fawcett & Taylor, 2012). To the extent that this mechanism is responsible for reducing the fidelity of long-term memory traces that defy the intention to forget, we expect fidelity to be relatively worse for retrieved targets from no-think trials, compared with retrieved targets from think trials. Further research is required to verify this prediction.

### The Implications of Intentional Forgetting

Beyond basic laboratory applications, we speculate that processes similar to those that led to intentional forgetting in the current investigation are enacted to regulate thoughts or memories about unpleasant or adverse autobiographical events. As a coping mechanism, limiting the encoding of unwanted memories should reduce the likelihood of subsequently retrieving and dwelling on an unwanted experience while also reducing the vividness of that experience should it be accidentally retrieved in contradiction to the encoding intention. Viewed in this light, intentional forgetting—even when performed at encoding—might serve the adaptive function of maintaining good mental health by mitigating the impact of unwanted thoughts and freeing us to focus on more productive pursuits (see also, Fawcett et al., 2015).

However, while generally adaptive (Bjork, 1972), we anticipate that there are also circumstances in which the enactment of such intentions might prove detrimental. For example, in the case of eyewitness testimony, it is conceivable that individuals could be asked to retrieve information that they had earlier intended to forget. Our results lead us to surmise that eyewitnesses would be relatively unlikely to retrieve the memory. But even more concerning is that when they do successfully retrieve the memory, the accuracy of their report might suffer as a result of their earlier attempt at forgetting (as evidenced by our color judgment results). Indeed, such concerns surround recovered memories of abuse (for discussion, see Anderson & Huddleston, 2012; Gordon & Connolly, 2010). And while recent laboratory findings suggest that participants maintain at least a general representation of those events they attempt to forget (e.g., Fawcett et al., 2013a, 2013b), this conclusion follows from single-trial attempts at intentional forgetting. Given that retrieval attempts provide additional encoding opportunities (e.g., Buckner, Wheeler, & Sheridan, 2001), it is possible that multiple retrieval attempts might also provide multiple forgetting opportunities. If the effects of memory intentions are cumulative, memories that survive repeated attempts to forget might become increasingly inaccurate and vulnerable to misinformation (e.g., MacLeod & Saunders, 2008) with each unintended retrieval. If so, this implies that our color judgment task might severely *underestimate* the effects of forgetting on memory fidelity in the real world. Whether or not this proves to be the case, the current experiments do tell us that when forced to describe an experience that we have attempted to forget, our descriptions are likely to be less accurate/more variable than might otherwise be expected (see also, Catarino et al., 2015; Fawcett et al., 2013a, 2013b; Küpper et al., 2014; c.f., Joslyn & Oakes, 2005).

Our study also provides a crucial methodological link between characterizations of episodic memory on the one hand, and of autobiographical memory on the other hand. Whereas the study of episodic memory tends to emphasize the probability of memory retrieval—viz. the proportion of studied items that are recalled or recognized—the study of autobiographical memory tends to emphasize the qualitative aspects of retrieval, such as accuracy and vividness. By obtaining independent measures of the probability of retrieval and the fidelity of retrieved traces, our methods offer a unique way of bridging the study of these two types of long-term memory. To the extent that memory processes are general, attempts to intentionally forget autobiographical memories should likewise show effects of that intention on the probability and fidelity of subsequent retrieval. Our study provides the rationale for further investigations to explore this question and to also determine whether such impacts are modulated by other considerations (such as repeated recall attempts, misinformation, and emotion). Whether or not our results generalize from episodic memory to autobiographical memory, they provide solid evidence that memory intentions formed at encoding have wider-ranging effects than have hitherto been documented.

Our current findings also highlight important parallels between the nature of stored long-term memory representations and the formation of visual short-term representations. Indeed, by adopting a modeling approach developed for the study of visual short-term memory to the study of long-term episodic memory, we have highlighted commonalities in how unattended and unwanted memories are represented within and beyond short-term working memory. As we described earlier, Zhang and Luck (2008) demonstrated that uncued color swatches are represented in visual short-term memory less often and with lower fidelity than attended color swatches. Our current results extend these findings to show that the active expulsion of unwanted F items from short-term working memory causes these items to be represented in episodic long-term memory less often and with lower fidelity than R items. To the extent that encoding intentions are implemented through cognitively demanding attentional changes as we have described, these results taken together point to attention as a critical factor in shaping memory in both the short- and long-term. When attention is never fully allocated to an item or else is withdrawn from it, that item is less likely to be represented in visual short-term working memory and less likely to be represented in long-term episodic memory; moreover, when it is represented in either memory system, it is represented with less precision than if the item had been attended and rehearsed.

## Conclusion

Regardless of ultimate application or generalizability to other memory systems, our study is the first to show that the enactment of an intention to forget at encoding reduces the probability of later retrieval as well as the fidelity of those long-term memory traces that are retrieved. Whereas there is a tradition of measuring long-term differences in the probability of retrieving to-be-forgotten information, ours is one of very few studies to reveal differences in the fidelity of to-be-forgotten information that is ultimately retrieved. Showing that the intention to forget impacts not only the probability but also the fidelity of retrieval is important because it shows that an intention to forget impacts even those memories that defy the intention: Put differently, retrieved F items are distinct from retrieved R items. Thus, just as intentional forgetting is different from unintentional forgetting, intentional remembering is different from unintentional remembering.

## Supplementary Material

10.1037/xge0000128.supp

## Figures and Tables

**Table 1 tbl1:** Estimated Log-Transformed and Back-Transformed Study Phase Probe Reaction Time (RT) for Experiments 1–3 as a Function of Instruction (F, R)

	RT (log-scale)		RT (ms)	
	F	R	F	R
Experiment 1	6.34 [6.22, 6.45]	6.22 [6.11, 6.34]	564 [503, 632]	503 [450, 565]
Experiment 2				
Study	6.32 [6.24, 6.40]	6.18 [6.08, 6.29]	553 [512, 601]	485 [436, 537]
Baseline	6.11 [6.02, 6.19]	6.09 [5.99, 6.18]	448 [412, 488]	440 [398, 485]
Study − Baseline	0.21 [0.15, 0.27]	0.10 [0.02, 0.18]	105 [75, 135]	44 [9, 80]
Experiment 3				
Study	6.23 [6.16, 6.30]	6.12 [6.04, 6.20]	509 [473, 546]	455 [420, 491]
Baseline	6.00 [5.95, 6.06]	5.99 [5.93, 6.04]	407 [385, 430]	398 [374, 422]
Study − Baseline	0.22 [0.16, 0.29]	0.13 [0.05, 0.20]	101 [70, 133]	57 [28, 95]
*Note.* Parenthetical values represent the HDI_95%_.				

## Footnotes

1 Reaction times were log-transformed across all relevant analyses in the present experiments; this was necessary because our models were fit to the “raw” trial-by-trial responses rather than the aggregate subject-level scores typical of some other approaches.

2 We should note here that whereas we have employed “maximal” random structure whenever possible in our other models (see Appendix A), this was not possible in the analysis of color judgments in the present experiment or in Experiment 3. This is because in applying our model only to those trials for which participants recognize or recollect the study item, we are rejecting approximately half of the usable test trials, leaving insufficient data to estimate a random slope for our effect (although we still include a random intercept). Nonetheless, because we replicate our findings twice in the present work—and also because we replicate them once again using a different approach in Experiment 4—we feel that any concerns related to the underestimation of variance are unwarranted in the present case.

3 In this instance the difference between the R and F conditions is not equal to the difference between the reported estimates for those conditions; this is because we report median values for our estimates and—especially for cases where the data are not normal—the median of a difference score is not the same as the difference between the medians of the corresponding variables.

4 Recognizing recent challenges to the adequacy of the independent remember/know procedure (e.g., Ingram et al., 2012; Moran & Goshen-Gottstein, 2015), we also conducted an analogous model on the raw “know” responses for which the intercept was −0.92, *HDI*_*95%*_ [−1.12, −0.72], with a slope of 0.21 for F items, *HDI*_*95%*_ [−0.03, 0.45], and 0.07 for R items, *HDI*_*95%*_ [−0.20, 0.32]. According to this model, participants failed to discriminate between foils and study items with respect to know responses. Nonetheless, a highly similar pattern emerged: Participants were numerically more likely to “know” that either R (*M* = .30, *HDI*_*95%*_ [.24, .35]) or F (*M* = .33, *HDI*_*95%*_ [.28, .38]) items had been presented than to falsely report “knowing” that a foil (*M* = .28, *HDI*_*95%*_ [.24, .32]) had been presented, although performance was comparable for R and F items (*M*_*R-F*_ = −.03, *HDI*_*95%*_ [−0.09, 0.03]).

5 The exclusion of buffer trials in Experiments 2 and 3 allowed us to combine these data to determine whether the F > R difference occurred even for the initial study phase trial, when there was no memory load. This analysis was motivated by the possibility that slower RTs post-F than post-R instruction might not be due to greater effort associated with implementing the F instruction but due to retrieval and cumulative rehearsal of R items on F trials. As pointed out by Fawcett and Taylor (2012), there is no reason to expect that the retrieval of preceding R items on F trials would produce the interaction between directed forgetting and inhibition of return that was observed by Taylor (2005; Fawcett & Taylor, 2010; Taylor & Fawcett, 2011), impair incidental memory formation as demonstrated by Fawcett and Taylor (2012) or reduce color-naming interference as reported by Lee, Lee, and Fawcett (2013). Nevertheless, we elected to determine whether there was still an F > R difference in probe RTs when there were no preceding study trials and therefore no prior R items to retrieve and rehearse. When we restricted our analysis to probe RTs obtained on the very first study trial, the model demonstrated slower RTs for F trials (*M* = 6.26, *HDI*_*95%*_ [6.12, 6.40]) compared with R trials (*M* = 6.06, *HDI*_*95%*_ [5.91, 6.21])—a difference of −0.21, *HDI*_*95%*_ [−0.41, −0.01]. We reach the same conclusion if we analyze baseline-corrected RTs instead. Thus, these data demonstrate that longer probe RTs post-F than post-R is not due to differences in the tendency to retrieve and cumulatively rehearse R items from preceding study trials.

6 We opted to use the formula provided by van den Berg and Ma (2014) rather than the more simplistic approximation of taking the square-root of the inverse (i.e., [1/ κ]^0.5^; e.g., Williams et al., 2013) because the latter approach becomes unstable for conditions where κ is low.

7 It is worth noting that the degree of encoding variability included in our variable-precision mixture model was itself modeled as a parameter, meaning that in the event that no encoding variability was evident, the encoding variability term would naturally tend toward 0 such that the model as a whole would become equivalent to a fixed-precision model.

8 Experiments 2 and 3 were not designed with mixture modeling in mind, including fewer trials and requiring participants to parse trials into those that were or were not recognized rather than estimating mathematically this value. Nonetheless, to connect the present results to those from the preceding experiments, the color judgments from Experiments 2 and 3 were combined and the same mixture model was applied. Collapsing these experiments was deemed necessary to compensate for the relatively small number of study items. To mirror the analyses from Experiments 2 and 3, we first applied our mixture model only to those trials for which participants indicated recognition of the study item: “yes,” “remember,” and “know” responses were collated and treated interchangeably. Due to the inclusion of only those trials for which participants reported recognition, we expected an effect of memory instruction on σ but little or no effect on ρ. This prediction was largely supported, with a roughly equivalent (*M* = .04, *HDI*_*95%*_ [−.33, .42]) back-transformed probability of recollecting R items (*M* = .62, *HDI*_*95%*_ [.23, .94]) compared with F items (*M* = .55, *HDI*_*95%*_ [.15, .94]), but greater variability observed for F items (*M* = 123.78°, *HDI*_*95%*_ [94.37°, 154.50°]) than R items (*M* = 85.69°, *HDI*_*95%*_ [52.30°, 105.92°]), with a median difference of 39.09°, *HDI*_*95%*_ [8.28°, 69.47°]. These findings provide strong, converging evidence for the claims made during the preceding experiments. We also attempted to apply the same mixture model to the combined data from Experiments 2 and 3 irrespective of the test phase responses. As in the model reported for the data from Experiment 4, we once again predicted an effect of memory instruction for both σ as well as ρ. The outcome of this mixture model generally supported the conclusions from Experiment 4, with a higher back-transformed probability of recollecting R items (*M* = .36, *HDI*_*95%*_ [.06, .80]) compared with F items (*M* = .30, *HDI*_*95%*_ [.03, .78]), and greater variability observed for F items (*M* = 127.59°, *HDI*_*95%*_ [86.78°, 161.68°]) than R items (*M* = 103.56°, *HDI*_*95%*_ [58.21°, 134.70°]). However, this model exhibited greater uncertainty in the estimated parameters and neither the effect of memory instruction on ρ (difference = .05, *HDI*_*95%*_ [−.36, .41]) nor on σ (difference = 24.23°, *HDI*_*95%*_ [−9.94°, 61.79°]) excluded 0. However, if the posterior distribution from Experiment 4 were used to inform the priors when modeling Experiments 2 and 3 as is often advocated (e.g., Kruschke, 2014, pp. 17–18), these effects are estimated with greater certainty, resulting in now credible differences of .11, *HDI*_*95%*_ [.03, .20], and 11.66°, *HDI*_*95%*_ [4.25°, 19.17°], for ρ and σ, respectively.

## A Methodological Details

Experiments 1, 2, and 3 employed fully Bayesian multilevel models analogous to the linear mixed-effects models endorsed by Dixon (2008). Our models were implemented using the *Stan* modeling language (Stan Development Team, 2013) rather than using the *lme4* package (Bates, Maechler, Bolker, & Walker, 2015). Except where otherwise specified, each of the reported models employed the “maximal” random structure justified by the model in question as recommended by Barr, Levy, Scheepers, and Tily (2013). Although details pertaining to the estimated random effects are not reported here, those values as well as further details pertaining to our models are available upon request from the first author (for a tutorial, see Sorensen & Vasishth, 2014).

For each of our models, efforts were made to ensure that suitably uninformative priors were placed upon each of our parameters and sensitivity analyses were carried out to ensure our findings remained unchanged across a range of alternatives. Each parameter estimate is reported in text along with a range indicating the corresponding highest-density interval (HDI; Kruschke, 2014). The HDIs summarize the most credible values of that parameter given the mathematical combination of the prior beliefs incorporated into our model for those parameters and the observed data. In this manner the HDIs can be interpreted intuitively (e.g., we are 95% confident that the true value rests within this range), and further probabilistic statements can be derived from the posterior as necessary (e.g., 75% of the credible values fell above 0 or equivalently we are 75% confident that the true value of this parameter rests above 0).

We conducted our models using a variant of an iterative technique known as Markov Chain Monte Carlo (MCMC) sampling (see Hoffman & Gelman, 2014). Such an approach iteratively polls possible parameter values until it converges upon those model parameters that optimally represent the data. This approach often requires a substantial number of iterations before the model converges upon the most credible solution; we erred on the side of caution and included a large number of iterations for each model. These iterations were split among four independent “chains” with random starting points. Each chain was allowed to converge independently, representing an independent replication of the model itself. The purpose of including independent chains is to ensure that the model reliably converges on the same parameters. However, because each chain is initialized with random starting parameters, they require a certain number of iterations before the optimal solution is reached—after which the posterior distribution is sampled directly. To ensure exclusion of this “burn-in” or “warm-up” period preceding convergence, we discarded the initial samples from each chain prior to collapsing the chains for analysis. With this procedure in mind, our models included chains of 20,000 iterations each (80,000 in total) with a burn-in period of 5,000 iterations per chain (20,000 total) resulting in 60,000 usable samples. Convergence was tested via visual inspection of the chains and also using the R-hat statistic (in all cases R-hat ≈ 1 and N_Effective_ > 1,000, indicating convergence; Gelman et al., 2014; Gelman & Hill, 2007; also discussed on p. 511 of Kruschke, 2010).

Finally, we evaluated the “fit” of our models using a combination of model comparison and posterior predictive checks. Model comparison was undertaken on the basis of the Watanabe-Akaike Information Criterion (WAIC) as well as Leave-One-Out cross-validation with Pareto Smoothed Importance Sampling (LOO-PSIS) using version 0.1.2 of the *loo* package implemented in R as described by Vehtari, Gelman, and Gabry (2015). These metrics provide an estimate of the out-of-sample predictive accuracy of our model and have been transformed to the deviance scale (i.e., lower values indicating better model performance) for reporting purposes. Posterior predictive checks were calculated following the general procedure recommended by Gelman et al. (2014, see Chapter 6; see also, Gelman, 2013). This process involves using the posterior distribution of our fitted model to simulate a large number of hypothetical data sets representing credible performance in our task should it be replicated. Presuming that the model in question is a good representation of performance in our task the simulated data should resemble the observed data. In addition to visual comparison of the simulated and observed data, we also calculated a posterior predictive *p*-value to evaluate the degree to which our fixed- and variable-precision mixture models captured the “peak” of the density curve observed for our data. This specific test quantity was motivated by the observation that mixture models of the sort reported in text tend to erroneously underestimate the peak of the density function in visual short-term memory tasks, resulting in structured residuals (e.g., van den Berg et al., 2012). To provide a test of this possibility we calculated the density of each simulated data set and compared the maximal value (i.e., the “peak”) against the value within the observed data set. An optimal fit would predict the observed data to be close to the center of the distribution of simulated values (i.e., with approximately 50% of the simulated values demonstrating a higher or lower peak). Our models captured the peak of the F trials quite well. Through simulation, we estimated the probability of a predicted value equal to or higher than the observed peak density for the F trials to be approximately 33% for our fixed-precision mixture model and 43% for our variable-precision mixture model. For the R trials the predicted densities tended to underestimate slightly the observed peak density. However, the peak remained well within the credible values predicted by our models. Through simulation, we estimated the probability of a predicted value equal to or higher than the observed peak density for the R trials to be approximately 14% for our fixed-precision mixture model and 17% for our variable-precision mixture model. Residual density plots are also provided in Supplementary Figure 1 depicting the subtraction between the density based upon the observed data and the simulated data for the variable-precision mixture model. Together, our models appear to adequately capture this feature of our data within a reasonable margin of error.

### Using Logistic Regression to Estimate Familiarity in an Independent Remember/Know Paradigm

In Experiment 3, each color wheel response within the test phase was preceded by an initial memory judgment in which participants indicated either that “no” they did not recognize the item, that they “knew” the item had been presented, or that they “remembered” the item having been presented. Whereas the primary purpose of the memory judgment was to permit analysis of the color judgments conditional on whether those items were accompanied by a self-reported recollective experience (as denoted by having “remembered” the item prior to color judgment), we nonetheless analyzed those judgments to determine whether intentional forgetting affected recollection and/or familiarity. However, as mentioned in text, these judgments involved a mutually exclusive response, meaning that as the proportion of “remember” responses increases in a given condition, the proportion “know” responses necessarily diminishes.

One method recommended for addressing the mutually exclusive nature of “remember” and “know” responses is to adopt the independent remember/know approach (e.g., Jacoby, Yonelinas, & Jennings, 1997). Using this approach, recollection is estimated as the probability of making a “remember” response but familiarity is estimated by dividing the proportion of “know” responses by 1 minus the proportion of “remember” responses:

$$F=\frac{p(K)}{1-p(R)}$$

However, rather than use this calculation to estimate familiarity, we chose to assess familiarity using a fully Bayesian analog in which we applied a logistic model only to those test phase trials for which a “remember” response was *not* made. To our knowledge, we are one of the first to adopt this particular approach and will therefore provide a brief explanation (see also, Fawcett & Ozubko, 2015).

Applying logistic regression to those test phase trials for which a “remember” response was *not* made can be shown to estimate the same underlying quantity as the traditional equation used to calculate familiarity. This is accomplished by algebraically reorganizing the preceding equation in terms of the number of each response type included in calculating the probability of “remember” or “know” responses. Specifically, the probability of either response type is equal to the relative frequency of that response divided by the sum of the relative frequencies of all possible responses. The preceding equation then becomes:

$$F=\frac{\frac{n_{K}}{n_{K}+n_{R}+n_{N}}}{\frac{n_{K}+n_{R}+n_{N}}{n_{K}+n_{R}+n_{N}}-\frac{n_{R}}{n_{K}+n_{R}+n_{N}}}$$

Wherein *n*_*R*_ refers to the number of “remember” responses, *n*_*K*_ refers to the number of “know” responses, and *n*_*N*_ refers to the number of “no” responses. Once the subtraction within the denominator is carried out we are left with the following equation:

$$F=\frac{\frac{n_{K}}{n_{K}+n_{R}+n_{N}}}{\frac{n_{K}+n_{N}}{n_{K}+n_{R}+n_{N}}}$$

This equation may be further simplified by replacing the division of the two fractions with multiplication by the inverse of the denominator. Doing so results in the following:

$$F=\frac{\left(n_{K})\times (n_{K}+n_{R}+n_{N}\right)}{\left(n_{K}+n_{N})\times (n_{K}+n_{R}+n_{N}\right)}$$

The final step is then to cancel out the terms common to both the numerator and the denominator, leaving us with the desired statement:

$$F=\frac{n_{K}}{n_{K}+n_{N}}$$

Put differently, traditional estimates of familiarity using the independent remember/know approach are equivalent to calculating the proportion of “know” responses after excluding trials for which a “remember” response has been made (as demonstrated by the absence of *n*_*R*_ in the denominator). With this fact in mind, our logistic model estimates the same underlying proportion as the traditional equations, but with the added flexibility of treating the data as truly binomial (e.g., Dixon, 2008; Jaeger, 2008) and permitting inclusion of complex random-effects structure (e.g., Wright, Horry, & Skagerberg, 2009). For further simulations proving the equivalence between these approaches, please contact the first author.

### Testing the Variable-Precision Mixture Model Against Simulated Data

Simulations were undertaken for the mixture models reported in Experiment 4 to ensure that the parameter estimates produced by these models were unbiased. These simulations were motivated by recent concerns that a mixture model might over- or under-estimate the model parameters (especially σ) when applied to a relatively small number of trials (e.g., Anderson & Awh, 2012). This concern might be viewed as especially relevant in our case: In the visual short-term memory literature, participants often complete a large number of trials (e.g., often 100 trials or more per condition) whereas the current investigation was much more constrained (i.e., 15 R and 15 F trials in Experiments 2 and 3 and 60 R and 60 F trials in Experiment 4). However, it is important to consider that the models employed in the present experiment differ from many more common variants in that our parameters were estimated hierarchically with subject treated as a random effect. We expected this hierarchical approach to insulate us against any biases introduced by the small number of trials within each condition. Our simulations supported this conviction.

We first simulated data drawn from the mixture of a von Mises distribution and a uniform distribution calibrated to approximate the structure (i.e., number of participants and trials per condition) and performance observed in Experiment 4. In these simulations, we assumed an effect of memory instruction for both σ and ρ of similar magnitude to what had been observed. We then fit our mixture model to these data and compared the recovered parameters against the known population parameters used to generate the data. This process was repeated 100 times so that we could then investigate the distribution of each parameter for signs of bias. We repeated this process once more assuming population parameters similar to those observed for the analysis of the combined data from Experiments 2 and 3 (see Footnote 8). These simulations are summarized in Supplementary Table 1. As depicted in that table, the models meant to emulate the data from Experiment 4 produced largely unbiased estimates for both σ and ρ and likewise for the effect of memory instruction on σ and ρ. These models also exhibited a great degree of statistical power: Across our simulations we observed a credible effect of memory instruction for σ or ρ in >90% of our samples.

The models meant to emulate the combined data from Experiments 2 and 3 (see Footnote 8) also performed reasonably well, with the exception that the effect of memory instruction on σ was underestimated by almost 50%; we attribute this to the especially small number of items per participant along with relatively poor memory performance, resulting in few trials for which the item was recollected and could therefore be used to model σ. We suspect that this produced a greater reliance on our skeptical priors, which would then push the effect towards 0. This means that those particular models are likely to be conservative with respect to this parameter. Further, the models for the combined data from Experiments 2 and 3 exhibited a distinct lack of statistical power: Although the estimated effects of memory instruction on σ and ρ were in the correct direction for 96% and 95% of the simulated models, respectively, these estimates were characterized by a great deal of uncertainty, and therefore only 3% of the models produced a credible difference for σ and none of the models produced credible differences for ρ.

Having evaluated our models for bias when memory instruction was known to impact σ and ρ, we also conducted the same simulations after setting the true population difference between our conditions to 0 for each parameter. These models would therefore reveal biases in the estimation of our effects when no true differences were present in the population from which the data were simulated. These simulations are summarized in Supplementary Table 2. As depicted in that table, neither the model based upon Experiment 4, nor the model based upon the combined data from Experiments 2 and 3 exhibited any evidence of bias, with parameter estimates close to the true population values. Further, credible differences were observed for σ or ρ in <5% of all simulations for Experiment 4 and in none of the simulations for Experiments 2 and 3—indicating an extremely low incidence of false alarms.

Taken together, these simulations permit us to interpret the models provided in text without fear of contamination due to biases introduced by our experimental design or modeling approach (the lone exception being that the mixture models applied to the combined data from Experiments 2 and 3 and reported in Footnote 8 are likely to be underpowered and to underestimate the magnitude of the effect of memory instruction on σ, if present; as noted in Footnote 8, using the posterior estimates from Experiment 4 to inform the priors for our exploratory analyses of the combined data from Experiments 2 and 3 provides a substantial reduction in the uncertainty of the parameter estimates derived from that model).

## B Supplementary Analyses

While we believe that the Bayesian models provided in text are more appropriate for the present data than their Frequentist counterparts, we nonetheless recognize that not all readers will be familiar or comfortable with our chosen analytic approach. We have therefore reanalyzed our core findings—where possible—using more traditional techniques. Absolute estimates for any given parameter tended to vary between the Bayesian and Frequentist models—e.g., due to rounding error, differences in model implementation or Bayesian shrinkage (e.g., see Sutton & Abrams, 2001)—but substantive conclusions are in all cases consistent with the models provided in text. In some cases, no “standard” equivalent exists for the analysis in question (e.g., the von Mises models reported for Experiments 2 and 3 or the mixture model reported in Experiment 4) and therefore those analyses were excluded from this appendix. Except where otherwise noted, data were pre-processed in the same manner as described in text.

### Experiment 1

#### Recognition Phase

The percentage of “hits” and “false alarms” made to studied and foil items during recognition phase trials were used to calculate *d*′. These *d*′ values were then analyzed as a function of memory instruction (R, F) using a repeated-measures ANOVA. This analysis revealed greater sensitivity to R items (*M* = 0.58, *SD* = 0.47) than to F items (*M* = 0.26, *SD* = 0.36), *F*(1, 19) = 19.35, *MSe* = 0.05, p < .001, η_G_^2^ = .132, with both significantly greater than 0 (both *p*s < .01). Response bias (C) could not be compared due to the use of a common false alarm rate.

#### Study Phase

Mean log-transformed reaction times for responses made within 100 ms and 2,000 ms of target onset were analyzed as a function of memory instruction (F, R) using a repeated-measures ANOVA. Responses were significantly longer following F instructions (*M* = 6.34, *SD* = 0.24) than following R instructions (*M* = 6.22, *SD* = 0.25), *F*(1, 19) = 15.78, *MSe* = 0.01, *p* < .001, η_G_^2^ = .054. These values correspond to back-transformed RTs of 567 ms and 504 ms, respectively.

A similar ANOVA was carried out on the accuracy of the probe detection responses. Although we observed greater probe detection accuracy on F trials (*M* = 97.72%, *SD* = 8.28%) than on R trials (*M* = 94.09%, *SD* = 18.25%), this difference failed to reach significance, *F*(1, 19) = 2.27, *MSe* = 58.29, p = .148, η_G_^2^ = .017. Further, the numerical difference itself was driven primarily by a particularly inaccurate participant, and if removed, overall accuracy for the F trials (*M* = 99.52%, *SD* = 2.09%) was comparable to R trials (*M* = 98.09%, *SD* = 3.81%).

### Experiment 2

#### Recognition Phase

##### Recognition accuracy

As in Experiment 1, the percentage of “hits” and “false alarms” made to studied and foil items during recognition phase trials were used to calculate *d*′. These *d*′ values were then analyzed as a function of memory instruction (F, R) using a repeated-measures ANOVA. Sensitivity was greater to R items (*M* = 0.43, *SD* = 0.55) than to F items (*M* = 0.20, *SD* = 0.59), *F*(1, 23) = 6.64, *MSe* = 0.10, p =.017, η_G_^2^ = .043. Whereas performance for R items was significantly above chance, *t*(23) = 3.87, *p* < .001, this comparison failed to reach significance for F items, *t*(23) = 1.65, *p* = .113. Response bias (C) could not be compared due to the use of a common foil false alarm rate for R and F trials.

##### Color judgments

The absolute degrees of error for each recognized item was analyzed as a function of instruction (F, R) using a repeated-measures ANOVA. Color judgments were significantly less accurate for F trials (*M* = 94.24°, *SD* = 23.56°) than for R trials (*M* = 69.41°, *SD* = 17.60°), *F*(1, 23) = 17.60, *MSe* = 420.39, *p* < .001, η_G_^2^ = .271.

#### Study Phase

As before, mean log-transformed reaction times for detection responses made within 100 ms and 2,000 ms of probe onset were analyzed as a function of memory instruction (F, R) using a repeated-measures ANOVA. Responses were significantly longer following F instructions (*M* = 6.32, *SD* = 0.16) compared to R instructions (*M* = 6.18, *SD* = 0.23), *F*(1, 23) = 19.17, *MSe* = 0.01, *p* < .001, η_G_^2^ = .101. These values correspond to back-transformed RTs of 554 ms and 485 ms, respectively.

We repeated these analyses using performance in the within-subject control task as a subtractive baseline. Each participant’s aggregate performance in the control task was calculated, log-transformed and subtracted from the log-transformed RTs, separately for R and F trials. Analysis of these data confirmed that responses were significantly longer following F instructions (*M* = 0.21, *SD* = 0.13) than following R instructions (*M* = 0.10, *SD* = 0.19), *F*(1, 23) = 9.06, *MSe* = 0.02, *p* < .01, η_G_^2^ = .106. Planned comparisons against 0 indicated that responses were slower in both the study phase F trials, *t*(23) = 8.10, *p* < .001, and R trials, t(23) = 2.72, *p* = .012, compared with the control task.

Finally, the mean proportions of responses made within the response window were analyzed as a function of memory instruction (F, R) using a repeated-measures ANOVA. This time we observed lesser accuracy for F trials (*M* = 96.59%, *SD* = 11.53%) than for R trials (*M* = 98.11%, *SD* = 4.63%), although this difference once again failed to reach significance, *F*(1, 23) = 0.72, *MSe* = 38.33, *p* = .405, η_G_^2^ = .008. As in Experiment 1, a single inaccurate participant drove the numerical difference; once removed, performance was numerically identical between these conditions (both *M* = 98.81%).

### Experiment 3

#### Recognition Phase

To mirror the analyses presented in text, we analyzed the “remember” and “know” responses using separate ANOVAs as a function of item type (foil, F, R). For our purposes, the remember responses were scored in the usual manner (i.e., dividing the total number of remember responses by the total number of trials) whereas to permit remember and know responses to vary independently, the know responses were calculated using the independent remember/know method (see in text for details). Accordingly, the know responses were tabulated and divided by the total number of trials excluding those in which a remember response had been made—or equivalently by calculating the proportion of “know” after excluding trials for which a “remember” response had been made.

##### “Remember” responses

The proportion of “remember” responses varied significantly across item type, *F*(2, 70) = 21.03, *MSe* = 110.65, *p* < .001, η_G_^2^ = .158, such that participants reported greater recollection of R items (*M* = 31.14%, *SD* = 18.08%) than F items (*M* = 23.84%, *SD* = 15.39%), *t*(35) = 2.81, *p* = .008, and more recollection of F items than unstudied foil items (*M* = 15.08%, *SD* = 11.93%), *t*(35) = 3.79, *p* < .001.

##### “Know” responses

For the “know” responses, there was a significant effect of item type, *F*(2,70) = 7.62, *MSe* = 168.99, *p* = .001, η_G_^2^ = .084. However, participants exhibited a comparable degree of familiarity for R items (*M* = 44.16%, *SD* = 18.76%) as for F items (*M* = 44.79%, *SD* = 17.06%), *t*(35) = 0.19, *p* = .853, but still reported more “know” responses for R or F items compared to foil items (*M* = 34.13%, *SD* = 12.72%), both *p*s < .001.

##### Color judgments

We analyzed absolute degrees of error separately for the color judgments made following “remember” and “know” responses each as a function of instruction (F, R). Categorizing the data in this manner resulted in three datasets with empty or near-empty cells, resulting in their exclusion. For items receiving a “remember” response, performance was better for R trials (*M* = 68.71°, *SD* = 29.10°) than for F trials (*M* = 92.21°, *SD* = 33.60°; *F*(1,32) = 9.63, *MSe* = 945.51, *p* = .004, η_G_^2^ = .125); however, whereas the same numerical pattern emerged for those trials receiving a “know” response, the difference between R trials (*M* = 86.12°, *SD* = 28.27°) and F trials (90.17°, *SD* = 26.15°) was not significant, *F*(1,32) = 0.34, *MSe* = 802.08, *p* = .565, η_G_^2^ = .005.

#### Study Phase

Mean log-transformed reaction times for responses made within 100 ms and 2,000 ms of target onset were analyzed as a function of memory instruction (F, R) using a repeated-measures ANOVA. Responses were significantly longer following F instructions (*M* = 6.23, *SD* = 0.21) than following R instructions (*M* = 6.12, *SD* = 0.22), *F*(1, 35) = 9.97, *MSe* = 0.02, *p* < .01, η_G_^2^ = .067. These values correspond to back-transformed RTs of 508 ms and 455 ms, respectively.

We repeated these analyses using performance in the within-subject control task as a subtractive baseline. Each participant’s aggregate performance in the control task was calculated, log-transformed and subtracted from the log-transformed RTs analyzed above. This process was conducted separately for R and F trials, even though the memory instructions were meaningless in the control task. Analysis of these data confirmed that responses were significantly longer following F instructions (*M* = 0.22, *SD* = 0.19) than following R instructions (*M* = 0.13, *SD* = 0.21), *F*(1, 35) = 4.73, *MSe* = 0.03, *p* = .0365, η_G_^2^ = .050. Planned comparisons indicated that responses were slower in both the study phase F trials, *t*(35) = 7.15, *p* < .001, and R trials, *t*(35) = 3.74, *p* < .001, than in the control task.

Finally, the mean proportions of responses made within the response window were analyzed as a function of memory instruction (F, R) using a repeated-measures ANOVA. As in Experiment 2, we observed lesser accuracy for F trials (*M* = 95.71%, *SD* = 12.19%) than for R trials (*M* = 96.96%, *SD* = 6.87%), although this difference was now very small and once again failed to reach significance, *F*(1, 35) = 0.67, *MSe* = 42.86, *p* = .419, η_G_^2^ = .004.

### Experiment 4

Because there is no “standard” equivalent for the mixture model reported in text, no data for this experiment were reanalyzed.
