# Supplementary material for: The Representational Consequences of Intentional Forgetting: Impairments to Both the Probability and Fidelity of Long-Term Memory
Source: J Exp Psychol Gen. 2016 Jan;145(1):56–81. doi: 10.1037/xge0000128 (PMC4694085; doi:10.1037/xge0000128)

Supplementary Table 1.

Population parameters and mean estimated model parameters for 100 simulated datasets based upon the mixture models of the data from Experiment 4 or of the combined data from Experiments 2 and 3 (see Footnote 8) when assuming an effect of memory instruction on σ and ρ. Difference scores were calculated such that positive values indicate a difference in the direction predicted in-text. Parenthetical values represent the standard deviation.

	**Population Parameters**		**Estimated Parameters**	
	σ	*ρ*	*σ*	*ρ*
**Experiment 4**				
Overall	33	.57	32.99 (1.52)	.58 (.03)
Forget	37	.49	37.53 (2.63)	.50 (.04)
Remember	29	.65	29.24 (1.42)	.66 (.03)
Difference	8	.16	8.29 (2.71)	.16 (.04)
% Credible Difference	–	–	93%	96%
**Experiments 2 and 3**				
Overall	118	.34	118.68 (6.09)	.37 (.07)
Forget	129	.30	125.93 (6.88)	.34 (.07)
Remember	104	.37	110.84 (8.64)	.40 (.08)
Difference	25	.07	15.08 (9.64)	.06 (.04)
% Credible Difference	–	–	3%	0%

Supplementary Table 2.

Population parameters and mean estimated model parameters for 100 simulated datasets based upon the mixture models of the data from Experiment 4 or of the combined data from Experiments 2 and 3 (see Footnote 8) when assuming that there is no effect of memory instruction on σ and ρ. Difference scores were calculated such that positive values indicate a difference in the direction predicted in-text. Parenthetical values represent the standard deviation.

	**Population Parameters**		**Estimated Parameters**	
	σ	*ρ*	*σ*	*ρ*
**Experiment 4**				
Overall	33	.57	32.69 (1.39)	.57 (.03)
Forget	33	.57	32.68 (1.78)	.57 (.03)
Remember	33	.57	32.76 (1.73)	.57 (.03)
Difference	0	0	-0.08 (2.11)	0 (.04)
% Credible Difference	–	–	1%	4%
**Experiments 2 and 3**				
Overall	118	.34	121.52 (6.53)	.36 (.05)
Forget	118	.34	121.60 (7.89)	.36 (.05)
Remember	118	.34	121.33 (7.54)	.36 (.05)
Difference	0	0	0.27 (8.19)	.00 (.04)
% Credible Difference	–	–	0%	0%

Supplementary Figure 1.

Residuals – defined as the subtraction between the density of the observed and the simulated data for each value of angular error – as a function of memory instruction and signed angular error for the variable-precision mixture model reported in Experiment 4; shaded regions represent the 95% HDI.

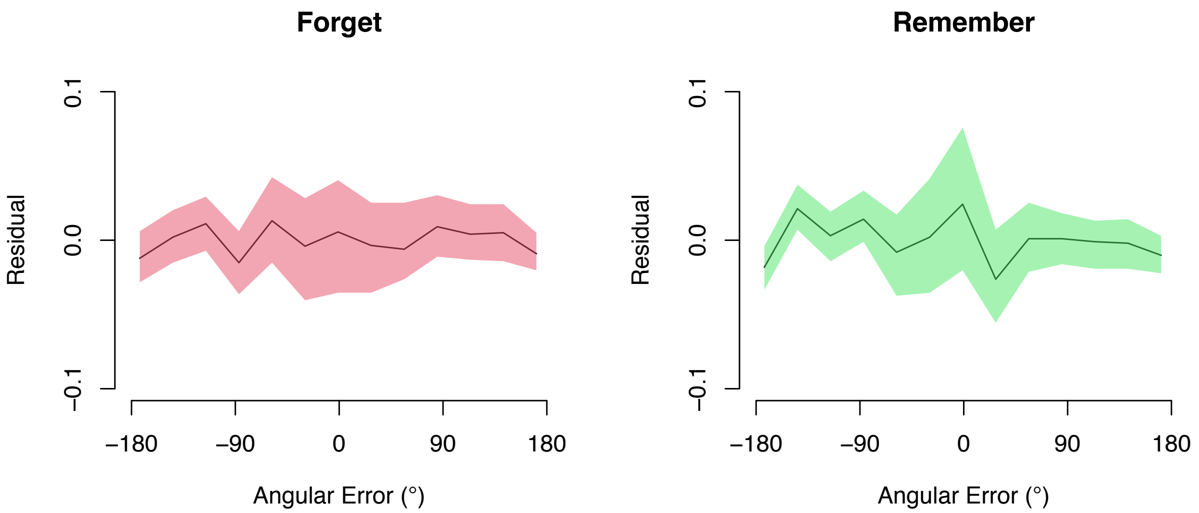

Supplement: Supplementary file 1 [file XGE-2014-0714R2-Forgetting_In_Color_Final_OnlineSupplement.pdf]
